# Characterization of regional differences in resting-state fMRI with a data-driven network model of brain dynamics

**DOI:** 10.1126/sciadv.abq7547

**Published:** 2023-03-17

**Authors:** Viktor Sip, Meysam Hashemi, Timo Dickscheid, Katrin Amunts, Spase Petkoski, Viktor Jirsa

**Affiliations:** ^1^ Aix-Marseille Université, INSERM, Institut de Neurosciences de Systèmes (INS), Marseille, France.; ^2^ Institute of Neuroscience and Medicine (INM-1), Research Centre Jülich, Jülich, Germany.

## Abstract

Model-based data analysis of whole-brain dynamics links the observed data to model parameters in a network of neural masses. Recently, studies focused on the role of regional variance of model parameters. Such analyses however necessarily depend on the properties of preselected neural mass model. We introduce a method to infer from the functional data both the neural mass model representing the regional dynamics and the region- and subject-specific parameters while respecting the known network structure. We apply the method to human resting-state fMRI. We find that the underlying dynamics can be described as noisy fluctuations around a single fixed point. The method reliably discovers three regional parameters with clear and distinct role in the dynamics, one of which is strongly correlated with the first principal component of the gene expression spatial map. The present approach opens a novel way to the analysis of resting-state fMRI with possible applications for understanding the brain dynamics during aging or neurodegeneration.

## Teaser

Machine learning for dynamical system discovery reveals heterogeneity across brain regions following structural variations.

## INTRODUCTION

One avenue for analysis of resting-state functional magnetic resonance imaging (fMRI) is the use of computational models of large-scale brain network dynamics (*1*, *2*). A general goal of this approach is to relate the observed brain activity to the dynamical repertoire of the computational model, possibly via identification of optimal model parameters, leading to a better mechanistic interpretation of the observations. One class of these computational models is network-based models, where the nodes represent brain regions and the edges represent the structural connections between them. These models can be constrained by individual brain imaging data; typically, the diffusion-weighted imaging data are used to estimate the edge weights. The local dynamics of brain regions is represented by the so-called neural mass models: low-dimensional models of neuronal population activity.

When linking the models with the observations, until recently, studies focused only on a small number of parameters because of the computational costs associated with the exploration of a high-dimensional parameter space. Typically, these would be the parameters affecting the network dynamics globally, such as a parameter scaling the strength of all connections in the network. In recent years, however, several works used the whole-brain modeling framework to explore the role of spatial heterogeneity of model parameters. Specifically, the studies found that the whole-brain models can better reproduce the features of resting-state fMRI when the regional variability is constrained by the MRI-derived estimates of intracortical myelin content (*3*), functional gradient (*4*), or gene expression profiles (*5*), and similar regional variability was found even without prior restrictions (*6*).

Neural mass models used in these studies [such as the dynamic mean field model of conductance-based spiking neural network (*7*) or Hopf bifurcation model of neural excitability (*8*)] are usually derived through a series of major simplifications to arrive at simple, low-dimensional models of neural dynamics. It can thus be questioned to what degree the dynamical structure embodied in these models is sufficient to capture the essential elements of the neural dynamics manifesting in the observed data. Would two different neural mass models lead to the same conclusions, or do the results strongly depend on the exact model form? Such questions are not yet sufficiently answered.

The recent advancements in dynamical system identification open up new possibilities in this direction and raise the question of whether a data-driven approach can be applied in the context of whole-brain modeling: Can we learn a dynamical system representing a neural mass at each node of a large-scale brain network? Such approach would allow us to side-step the issue of reliance on a specific neural mass model, which lie at the heart of the large-scale modeling, and instead extract this model directly from the functional data.

**Fig. 1.**
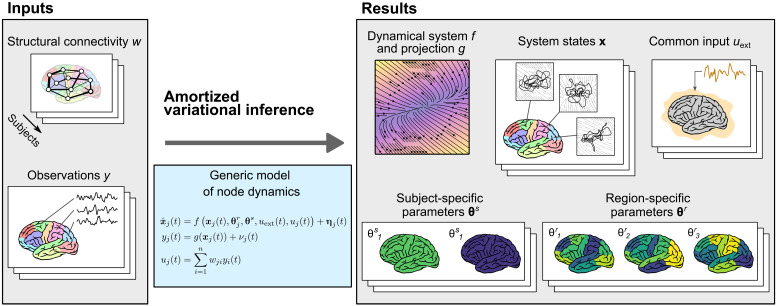
Conceptual overview of the method. The method allows us to perform a parameter inference for network models of brain dynamics, where the dynamical model of every node (or brain region) is initially unknown. As input (left), it expects structural connectivity matrices *w* for cohort of multiple subjects and corresponding observations of brain activity *y* (such as parcellated resting-state fMRI). Constrained by the structure of a generic model of regional dynamics (middle), it learns the dynamical model of node dynamics *f* and the state-to-observation projection model *g*. The dynamical model *f* is shared for all subjects and regions, but it depends on subject-specific parameters **θ***^s^* and region-specific parameters **θ***^r^*. These, too, are inferred from the data, together with the hidden states of the system **x** and the subject-specific external input *u*_ext_, shared by all regions in a given subject. All of the system states, subject- and region-specific parameters, and the external input are inferred probabilistically as normal distributions, that is, we infer the mean and variance for each parameter.

Our goal in this work is to learn the model of whole-brain dynamics using the framework of network-based models (Fig. 1). That is, we assume the model form of connected neural masses, with the connection strengths derived from diffusion-weighted imaging. We however do not specify the functional form of the neural mass and instead wish to learn the neural mass model in a data-driven fashion from the observed dynamics in resting-state fMRI. Using this model, we want to learn its parameters varying across regions and subjects, which, together with the individual connectome networks, give rise to the differences in observed dynamics across regions and subjects.

Given that this approach avoids the assumptions on the specific form of the regional dynamics, the desired framework would have a potential to independently validate the methodologies used in the traditional approaches with predefined neural mass models (such as the assumptions on the nature of regional dynamics) and support their finding (such as on the role of regional heterogeneity in whole-brain dynamics). Eventually, such approach might lead to a new generation of more expressive, data-driven network models of large-scale brain dynamics.

At the core of this work is the problem of data-driven nonlinear dynamical system identification. Many approaches have been introduced in recent years and applied in various fields of physical and life sciences (*9*–*14*), including in neuroscience on all scales (*15*–*17*). A wide range of approaches were used for the representation of the dynamical systems and for the inference methods: Brunton *et al.* (*9*) introduced the sparse identification of nonlinear dynamics (SINDy) method, which uses sparse regression to find the coefficients for a predefined library of nonlinear functions, leading to ordinary differential equations (ODEs) in human-readable form. Some authors approximated the nonlinear systems through blending locally linear dynamics; among those approaches is the switching linear dynamical system trained with message passing algorithm (*10*, *13*) or the method learning the position of fixed points and the associated Jacobians with variational inference (*11*). Other approaches include recurrent neural network with piecewise linear activation trained with the expectation-maximization algorithm (*14*, *16*). However, others relied on variational autoencoder architecture, representing the dynamical system with recurrent neural networks (*15*) and including a hierarchical parameterization of the nonlinear systems (*12*). These approaches differ in terms of computational costs, quality of resulting reconstructions, complexity of the implementation, and interpretability of the resulting system.

Our problem has several specifics that preclude straightforward adoption of the existing methods and motivate the development of the method presented here:

1) We wish to operate within the framework of network-based models of large-scale brain dynamics, meaning that we wish to incorporate the known structural connectivity in the whole-brain model. The method thus has to allow for this prespecified network connectivity.

2) We are interested in noise-driven systems, that is, systems described by stochastic differential equations rather than deterministic ODEs. Some of the existing methods focus only on deterministic ODEs and incorporate only observation noise, but not the noise in the underlying system.

3) It is the desired parameterization of the neural masses with regional and subject parameters. When faced with a problem of system identification for multiple related systems, many of the existing methods do not provide a way to share the learned dynamics; rather, the systems are inferred independently. Here, we wish to infer only one dynamical system with the intersubject and interregional variations represented via the regional and subject parameters only.

4) It is the problem of partial observations. It is sometimes assumed that all state variables are observed, simplifying the inference problem greatly. We assume that we have only one-dimensional (1D) observation of the regional dynamics available (such as the regional fMRI time series), meaning that multiple system states are hidden and need to be inferred as well.

To tackle this problem, we use the framework of amortized variational inference or variational autoencoders (*18*), inspired, in particular, by its application for inferring neural population dynamics (*15*) and for dynamical systems with hierarchical structure (*12*). Briefly, our system is composed of an encoding network, mapping the observed time series to the subject- and region-specific parameters and to the trajectory in the source space, a neural network representing the dynamical system, and the observation model acting as the decoder from the source to the observation space. These are jointly trained to maximize the evidence lower bound (ELBO) so that the predictions of the trained model closely resemble the original data.

In this work, we test our method on two synthetic datasets, generated with the two models commonly used in large-scale brain modeling: the mean field model of conductance-based spiking neural network, or mean field model for short (*7*), and the Hopf bifurcation model (*8*). For both test cases, we use a cohort of eight subjects with realistic structural connectomes and with model parameters varying across subjects and brain regions. We show that the trained generative model can reproduce many features of the original dataset and demonstrate that the method can extract regional and subject-specific parameters strongly related to the original parameters used for the simulation.

Last, we apply the method to resting-state fMRI data of 100 subjects from the Human Connectome Project (HCP) (*19*). We find that the inferred dynamics can be described as noisy fluctuations around a single fixed point, both on the node and network level. The method reliably discovers three regional parameters with clear and distinct function on the dynamics, one of which is strongly correlated with the first principal component of the gene expression spatial map. We further find that the functional connectivity (FC) is reproduced only partly and relies heavily on the external input shared by all regions and only to a small extent on the network interactions.

## RESULTS

The results section consists of three parts. In the first part, we introduce the general ideas of the developed method; its detailed description can be found in Methods. In the second part, we validate the proposed method on synthetic data, that is, on data generated by computational models with known parameters. In the third part, we apply the method on resting-state fMRI data from human subjects and analyze the results.

### Amortized variational inference for networks of nonlinear dynamical systems

We follow the general framework of large-scale brain network modeling and assume that for a specific subject, the observations *y_j_*(*t*) of a brain region *j* are generated by a dynamical system

$${\dot{x}}_{j}(t)=f[x_{j}(t),{\text{θ}}_{j}^{r},{\text{θ}}^{s},u_{\mathrm{ext}}(t),u_{j}(t)]+{\text{η}}_{j}(t)$$ (1)

$$y_{j}(t)=g[x_{j}(t)]+\nu_{j}(t)$$ (2)

where **x***_j_*(*t*) ∈ ℝ*^n_s_^* is the state at time *t* and ${\text{θ}}_{j}^{r}\in R^{m_{r}}$ and **θ***^s^* ∈ ℝ^*m*_s_^ are the region-specific and subject-specific parameters.

The term *u*_ext_(*t*) is the external input, shared by all regions of a single subject, and

$$u_{j}(t)=\sum_{i=1}^{n}w_{ji}g_{c}[x_{j}(t)]$$ (3)

is the network input to region *j* with ${\left\{w_{ij}\right\}}_{i,j=1}^{n}$ being the structural connectome matrix of the network with *n* nodes. The functions *f*, *g*, and *g_c_* are initially unknown, and **η***_j_*(*t*) and ν*_j_*(*t*) are the system and observation noise, respectively (fig. S1A).

From the observed time series of multiple subjects, we wish to infer both the evolution function *f* and observation function *g*, which are the same for all subjects, as well as region- and subject-specific parameters ${\text{θ}}_{j}^{r}$ and **θ***^s^* and the time-dependent external input *u*_ext_. To do so, we adopt the general framework of amortized variational inference (*18*) with hierarchical structure in parameters (fig. S1B) (*12*). We consider the states **x***_j_*, the parameters ${\text{θ}}_{j}^{r}$ and **θ***^s^*, and the external input *u*_ext_ the latent variables and seek their approximate posterior distribution represented by multivariate Gaussian distributions. In the spirit of amortized variational inference, we do not optimize their parameters directly but through encoder functions *h*_1_, *h*_2_, *h*_3_, and *h*_4_, which transform the data to the latent variables (system states, regional and subject parameters, and external input, respectively).

For reasons of computational tractability, we take a strong assumption that the observation and coupling functions are identical, *g* ≡ *g_c_*. This allows us to effectively decouple the network problem to uncoupled regions with known network input, so we can consider the time series of one region of one subject as a single data point. We return to this choice and its possible implications in light of the results in Discussion.

We represent the nonlinear function *f* with a generic artificial neural network and function *g* as a linear transformation. The inference problem is ultimately transformed into the optimization of the cost function, ELBO, which is to be maximized over the weights of *f*, *g*, *h*_1_, *h*_2_, *h*_3_, and *h*_4_ and over the variances of the system and observation noise. After the optimization, we obtain the description of the dynamical system in terms of functions *f* and *g*, probabilistic representation of the regional and subject parameters ${\text{θ}}_{j}^{r}$ and **θ***^s^* and of the external input *u*_ext_, and projections of the observations in the state space **x***_j_*. The inferred parameters ${\text{θ}}_{j}^{r}$ and **θ***^s^* will not have a mechanistic meaning; however, they can provide a measure of (dis)similarity of the regions and subject and can be interpreted via the inferred dynamical system *f*.

### Validation on synthetic data

#### Evaluation workflow

**Fig. 2.**
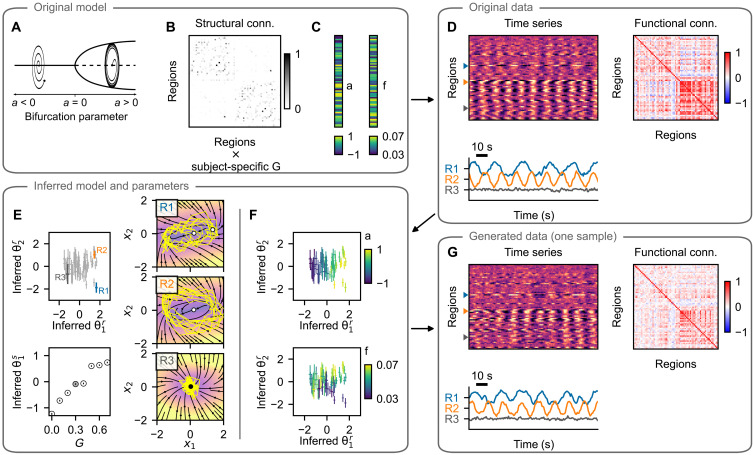
Hopf model test case: example subject. (**A**) The training data are simulated using a network model of brain dynamics, where, in each node, a Hopf neural mass model is placed. (**B**) The nodes are coupled through a connectome derived from diffusion-weighted imaging scaled by a subject-specific coupling parameter *G*. (**C**) The values of bifurcation parameter *a_i_* and intrinsic frequency *f_i_* vary across brain regions. (**D**) Time series generated with the original model with three examples (bottom) and the calculated FC (right). (**E**) Inferred regional parameters for all regions (top left; example nodes highlighted) and inferred subject-specific parameter (bottom left; in gray among parameters for all subjects in the dataset). The span of the crosses/lines corresponds to two SDs of the inferred Gaussian distribution. Bottom: Circles are added for visual aid because of the small SDs. The inferred dynamics in state space of the three example nodes are on the right. The vector field is evaluated assuming zero network input and the inferred parameters. Background color, velocity magnitude; yellow lines, exemplary simulated time series of the node activity; Black- and white-faced circles, stable and unstable fixed points. In the topmost panel, an unstable fixed point and a stable fixed point are visually overlapping. (**F**) Inferred regional parameters colored by the ground truth values of the bifurcation parameter *a_i_* (top) and frequency *f_i_* (bottom). The bifurcation parameter correlates with inferred $\theta_{2}^{r}$, while frequency correlates with $\theta_{1}^{r}$, but only for regions in the oscillatory regime, i.e., where *a_i_* > 0. (**G**) One example of the time series generated with the trained model and using the inferred parameters. Important features of the data are preserved both on the regional level (amplitude and frequency) and on the network level (FC).

**Fig. 3.**
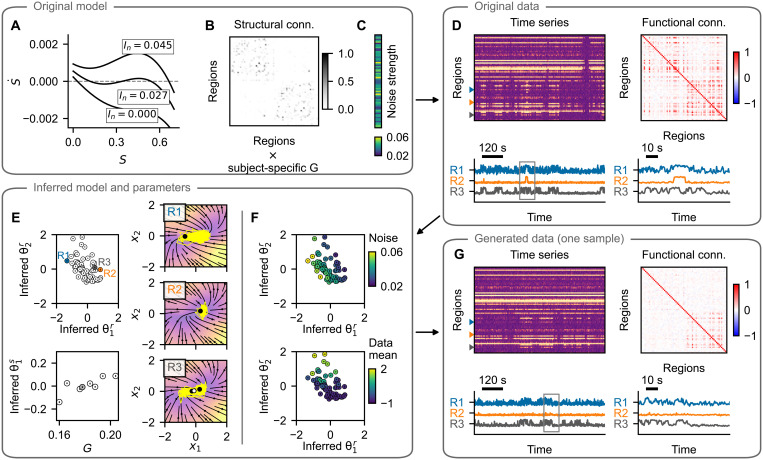
pMFM test case: example subject. Layout is the same as in Fig. 2. (**A** to **C**) The training data are simulated using a network of pMFM neural masses. Depending on the network input, these can be forced into the monostable regime (down- or up-state) or into the bistable regime. The dynamics is noise driven, with noise strength varying across regions. (**D**) Time series generated with the original model and the FC. Three examples are shown in the bottom panel, with a window of 100 s on the right. (**E**) Inferred regional parameters (top left) and subject-specific parameter. Circles are added for visual aid because of the small SDs. The inferred dynamics in state space of the three example nodes are on the right. The vector field is evaluated assuming network input *u* = 1 and using the inferred parameters. Background color, velocity magnitude; yellow lines, exemplary simulated time series of the node activity; black- and white-faced circles, stable and unstable fixed points. (**F**) Inferred regional parameters colored by the ground truth values of the noise strength parameter (top). The original parameter is stored along the diagonal of the inferred parameter space. Bottom: Coloring according to the mean of the original time series, which does not represent an original model parameter, rather a data feature. (**G**) One example of the time series generated with the trained model and using the inferred parameters. Region-specific features (switching between states and noisiness) are well preserved. Structure of the regional correlations is also reproduced, but the correlations are weaker compared to the original. Note that even if the regional time series exhibit signs of bistability (up- and down-state), this bistability may arise at the network level and not necessarily at the regional level, as evidenced by the phase plots in (E).

We test the proposed method on two synthetic datasets, where the data are generated by models commonly used in whole-brain modeling. First is the Hopf bifurcation model (*8*), shown in Fig. 2. That is a two-equation neural mass model, where, depending on the value of the bifurcation parameter *a_i_*, the dynamics is either noise driven around a stable fixed point (for *a_i_* < 0) or oscillatory with frequency *f_i_* (for *a_i_* > 0). In the synthetic dataset, these two parameters are randomly varied across regions. The second model is the parametric mean field model (pMFM) (*7*), shown in Fig. 3. That is an one-equation model, and depending on the network input, it can be pushed into monostable down- or up-state or a bistable regime. The switching between the states is noise driven, and we vary the noise strength across brain regions.

Both models are used to generate synthetic data for eight subjects, each with individual structural connectome containing 68 cortical regions of the Desikan-Killiany parcellation (*20*). The connectome is scaled by the global coupling strength *G*, which we set to increase linearly across subjects for the Hopf model or which we set to the optimal value (in terms of highest produced FC), different for every subject, with pMFM.

To establish the performance of the described method, we proceed as follows. First, we simulate the data with the original model and random values of regional parameters (Figs. 2D and 3D). Next, using the whole dataset of eight subjects, we train the model, obtaining at the same time the trained generative model described by the function *f* of the dynamical system, as well as the probabilistic representation of subject- and region-specific parameters (Figs. 2E and 3E). Taking random samples from the posterior distributions of the parameters and using random system and observation noise, we repeatedly generate new time series using the trained model (Figs. 2G and 3G).

**Fig. 4.**
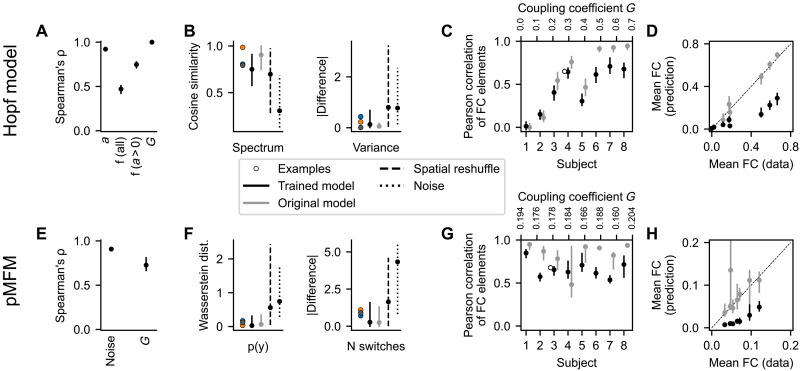
Quantitative evaluation of the synthetic test cases. Top row: Hopf model. Bottom row: pMFM. (**A** and **E**) Nonlinear correlation between the original parameters and the optimal projection of the inferred region-specific parameters (bifurcation parameter *a* and frequency *f* for Hopf model and noise strength for pMFM) and subject-specific parameter (coupling strength *G*). (**B** and **F**) Fit between the regional features of the original time series and those generated by the trained model. We show the cosine similarity of the time series spectra and the difference in variance for the Hopf model and Wasserstein distance of the distributions in the observation space and the difference in logarithm of number of switches for pMFM. These are evaluated for the examples from Figs. 2 and 3, all time series generated by the trained model, and the surrogates described in the main text. (**C** and **G**) Fit between the FC of the original and generated time series. (**D** and **H**) Mean value of nondiagonal elements of FC matrices. For both models, the correlation strength is underestimated, even if the structure is preserved. In all panels, the bars show the (5, 95) percentile interval with the dot representing the median value. The statistics are computed from 50 samples of the posterior distribution for eight subjects [grouped together in (A), (B), (E), and (F)] and 68 regions (for region-specific parameters and features). The statistics of the surrogate distributions using the original model are also calculated from 50 samples.

We evaluate the quality of the trained model on the basis of the following criteria. First, we establish whether the inferred parameters are related to the original parameters of the model (Figs. 2F, 3F, and 4, A and E). Second, we wish to evaluate whether the features of the generated time series resemble those of the original time series, both on the regional level (Fig. 4, B and F) and on the network level (Fig. 4, C, D, G, and H). We explicitly note that we evaluate this similarity using the time series sampled from the posterior predictive distribution (that is, the time series are generated with the inferred parameters **θ***^r^* and **θ***^s^* but using random system and observation noise) and not from the posterior distribution *q*(**x**∣**y**, **u**, **c**) from Eq. 26.

#### Inferred parameters correspond to the original model parameters

The example on Fig. 2F shows how the original regional parameters are linked to the inferred parameters **θ***^r^* for the Hopf model. The bifurcation parameter *a* maps to the inferred parameter $\theta_{1}^{r}$ (top), while the frequency *f* maps to $\theta_{2}^{r}$ (bottom). The latter is however true only for the regions in the oscillatory regime, i.e., with *a* > 0. That is not a deficiency of the proposed method: In the fixed-point regime the activity is mainly noise driven, and the value of the frequency parameter has small to negligible influence (see the example C on Fig. 2D). In other words, the parameter is not identifiable from the data. That is reflected in the inferred parameters. For the regions with *a* > 0 (or equivalently with $\theta_{1}^{r}>0$) the inferred parameters $\theta_{2}^{r}$ have low variance, and their mean maps to the original frequency parameter. For the regions with *a* < 0, however, inferred $\theta_{2}^{r}$ have high variance, close to the prior value 1, and overlapping distributions, indicating that not much information is stored in $\theta_{2}^{r}$ in this regime.

In addition, for the pMFM test case, the noise strength parameter is well identified (Fig. 3F); however, the second dimension of the region-specific parameter $\theta_{2}^{r}$ correlates with the mean of the regional time series. Presumably, this is so that the parameter $\theta_{2}^{r}$ can compensate for the weaker network coupling, which we discuss later. For both examples, the subject-specific coupling strength is mapped to the subject parameter **θ***^s^* (Figs. 2E and 3E, bottom).

The quantitative analysis of the goodness-of-fit is shown on Fig. 4 (A and E). To evaluate it, for each of the original parameters, we first identified the direction in the parameter space along which the parameter is stored by taking a linear regression of the posterior distribution means. Then, we repeatedly took samples from the posterior distributions of the parameters, projected them on the identified subspace, and calculated the nonlinear Spearman’s correlation coefficient ρ. For most parameters, the values are close to the optimal value of 1, indicating that the original parameters are indeed accurately recovered in the inferred parameters. The exception is the frequency *f* due to the above discussed nonidentifiability. If, however, we restrict the regions only to those where the bifurcation parameter is positive, then the correlation markedly increases, as expected on the basis of the discussed example. Further illustration of the parameter correlations and reported statistics is presented on figs. S2 and S3.

On fig. S4, we further evaluate how the goodness of fit changes with the increased coupling in the Hopf model. Presumably, as the coupling increases, the regional time series are more affected by the activity of the connected regions and less by its internal parameters, and it is thus more difficult to recover the original parameters from the data. That is the trend that we observe both for the bifurcation parameter *a* and frequency *f* of the nodes in oscillatory regime.

#### Trained model reproduces the features of regional time series

A crucial test of the trained model is an evaluation of whether the generated data resemble those used for the initial training. This resemblance should not be understood as reproducing the time series exactly because they depend on a specific noise instantiation, rather that the features that we consider meaningful should be preserved. For both test cases, we evaluate the similarity of the two features. For the Hopf model with its oscillatory dynamics, we evaluate the cosine similarity of the spectra of the original and generated time series and the difference between the variance of the time series because the variance differs greatly between the nodes in oscillatory and fixed-point regimes (Fig. 4B and fig. S5). For the pMFM, we compare the time series based on the distribution in the 1D observation space (that is, taking the samples collapsed across time) using the Wasserstein distance (also called Earth mover’s distance) of two distributions. The second feature of the pMFM time series is the log-scaled number of switches between the up- and down-state, capturing the temporal aspect of the switching dynamics (Fig. 4F and fig. S6).

We evaluate the measures for 50 different noise instantiations, leading to 50 different time series for each region, obtaining a distribution of goodness-of-fit metrics. The same metrics are also evaluated for three surrogates: First is the original computational model, run with different noise instantiations. That provides an optimistic estimate of what can be achieved in terms of goodness of fit, considering that the features will necessarily depend on the specific noise instantiation used in the initial simulations. The second surrogate is obtained by randomly reshuffling the original data between regions and subjects. The third surrogate is simply white noise with zero mean and variance equal to one (which, because of the data normalization, is equal to the mean and variance of the original dataset taken across all subjects and regions).

In most measures, the trained model performs comparably or slightly worse than the original model and markedly better than both the reshuffled surrogate and the noise surrogate (Fig. 4, B and F; numerical values and results of statistical tests are in table S2). The exception is cosine similarity of the spectra with the Hopf model. In this measure, the reshuffling surrogate achieves similar values as the trained model; this is due to the fact that many of the regions in the coupled networks oscillate on similar frequencies. Therefore, the time series of two randomly selected regions often reach high scores in cosine similarity of their spectra.

#### Functional network structure is reproduced but with lowered strength

Just as the well trained model should be able to reproduce the features of the original data on the level of single regions, it should also be able to reproduce the relevant features on the network level. Specifically, we evaluate how well the FC is reproduced. In general, FC quantifies the statistical dependencies between the time series from brain regions. While there are multiple ways to measure it, the most ubiquitous is the linear (Pearson’s) correlation of the time series, which we use here as well. This static FC captures the spatial structure of statistical similarities; however, it has its limitations. Notably, it ignores the temporal changes in FC structure (*21*, *22*).

The examples for both investigated models indicate that the FC structure is well reproduced but with lower strength, particularly in the case of pMFM example (Figs. 2G and 3G). This is further analyzed for all subjects on Fig. 4 and visualized on figs. S7 and S8. For the Hopf model, the coupling coefficient was increased between subjects. For low coupling values, the FC structure is not reproduced (as measured by Pearson correlation between the nondiagonal elements of original FC and trained model FC; Fig. 4C). That is however also true for the original model because of the FC elements being close to zero and noise dependent. For stronger coupling, the structure is preserved better, although the trained model plateaus around values of 0.7 for the correlation between the FC matrices, even when the correlations between the original model increases further. The comparison of the mean value of nondiagonal FC elements furthermore reveals that the strength of the correlations is considerably underestimated with the trained model (Fig. 4D). For the pMFM, the coupling coefficient was set to optimal value (in the sense of maximal FC), specific to each subject. In addition, there, we can see a well-reproduced structure of the correlations (Fig. 4G), although with reduced strength as well (Fig. 4H).

These results indicate that while the trained model can discover the existence of the network coupling, it systematically underestimates its strength. Given that in the pMFM, the strength of the network input can shift a single neural mass from the monostable down-state to the bistable regime and to monostable up-state, the underestimated coupling leads to the necessity of using the regional parameter to compensate for the missing coupling (Fig. 3F). Speculatively, this reduction in the network coupling strength contributes, to some degree, to the less-than-perfect recovery of the regional parameters because the features of the local dynamics are affected by the network interactions. For instance, in the Hopf model networks, the strongly coupled regions synchronize together; without the network coupling taken into account, the local frequency parameter might be considerably over- or underestimated.

#### Large perturbations of the connectome lead to reduced performance

To assess the influence of the inexact structural connectome on the goodness of fit, we have trained the model on the pMFM dataset with perturbed connectomes. That is, instead of the original connectivity matrix *W*, we have trained the model with *W*_ϵ_ = *W* + ϵ*A*, where *A* is the matrix with elements drawn from standard normal distribution and ϵ > 0 is the perturbation magnitude. In addition, we have also used a log-scaled connectivity matrix.

Figure S9 shows how the indicators of goodness of fit from Fig. 4 are modified by using these surrogate connectomes. High perturbation magnitudes reduce the recovery of regional and subject parameters (fig. S9, A and B), as well as the similarity of the generated FC (fig. S9E). The regional features, on the other hand, are reproduced similarly well even for large perturbations (fig. S9, C and D). Using the log-scaled connectome has a similar negative effect, although less pronounced.

### Application on human resting-state fMRI

We applied the developed method to human resting-state fMRI data obtained from the HCP (*19*). For 100 subjects, we have analyzed fMRI time series from one session (864 s at 0.72 sampling rate), processed with the HCP pipeline, further denoised by Diffuse Cluster Estimation and Regression (DiCER) method (*23*), and parcellated into 68 cortical regions of Desikan-Killiany parcellation (*20*). Where not mentioned otherwise, the results presented are obtained with model having state space dimension *n_s_* = 3 and regional parameter dimension *m_r_* = 3 and for the variant with external input and with standard preprocessing of the structural connectomes (see Methods for details). We have set the subject parameter dimension *m_s_* to zero because of the observed difficulties with the convergence of the subject parameters; these are illustrated on fig. S10.

**Fig. 5.**
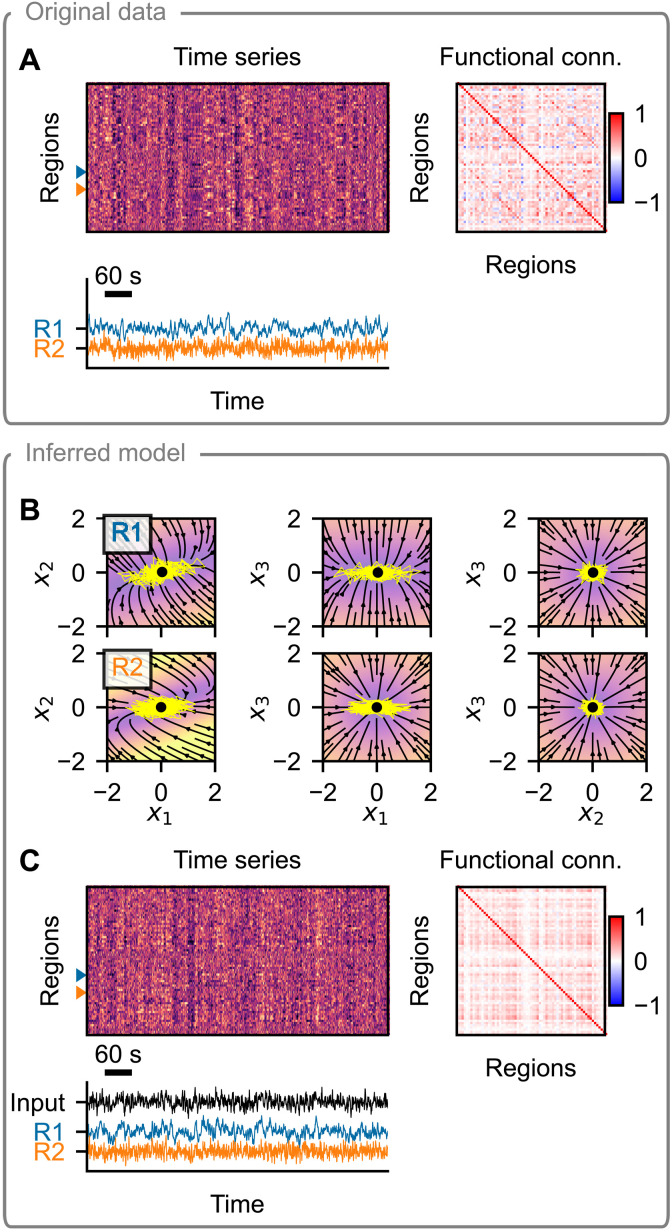
Example of model inference on a single subject from the HCP dataset. (**A**) Resting-state fMRI data (top left), corresponding FC (top right), and time series of two example regions (bottom). (**B**) Phase plane of the inferred dynamical system. Each panel shows a 2D projection of a 3D system, with the third variable set to the position of the fixed point (black dot). The vector field is evaluated assuming zero network input and using the inferred region-specific parameters. Background color represents the velocity magnitude; yellow lines are exemplary simulated time series of the node activity when embedded in a network. (**C**) Data generated by the inferred model. The regional parameters are set to those inferred, while the system and observation noise is random. The external input is generated randomly using the inferred parameters. Layout is the same as in (A).

We start this section with an example of the original data and the inference results for a single subject on Fig. 5. For this subject, the original time series exhibit moderately strong coupling, with widespread patterns visible in the processed fMRI data (Fig. 5A). We trained the model using the data from all of the 100 subjects but with randomly selected 20% of regional time series left out to assess possible overfitting (fig. S11). Upon the first inspection, the trained model relies on a single fixed point dynamics on node level and can reproduce several aspects of the original data well (Fig. 5C): The noisy nature of the regional time series and the presence or absence of the low-frequency oscillations are qualitatively similar to the original data. In addition, the widespread fluctuations are present and reflected in the FC. We evaluate these and further aspects in the next paragraphs.

#### Noisy dynamics around a single fixed point

**Fig. 6.**
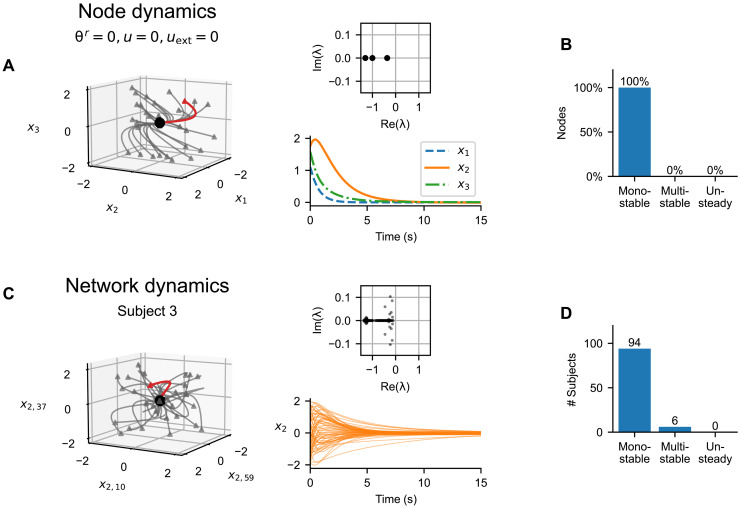
Discovered node and network dynamics. (**A**) Node dynamics for an uncoupled node with **θ***^r^* = 0, *u* = 0, and *u*_ext_ = 0. The left subpanel shows the trajectories from random initialization points (triangles) to the stable fixed point (black point). The red trajectory is visualized on the bottom right subpanel; the top right shows the eigenvalues of the system at the fixed point. (**B**) Inferred dynamical regimes of the uncoupled nodes. We analyzed the discovered system for 680 inferred parameter configurations and nine combinations of *u* and *u*_ext_ (see the main text). (**C**) Network dynamics for a single subject using the known structural connectome and inferred regional parameters. The left subpanel shows the trajectories from random initialization points (triangles) converging to the stable fixed point (black point) in the absence of system noise in three selected dimensions (*x*_2_ in three nodes). The red trajectory is visualized on the bottom right subpanel; the top right shows the eigenvalues of the system at the fixed point. (**D**) Inferred dynamical regimes of the subjects. We analyzed the network dynamics using the inferred regional parameters and the known structural connectomes. For each subject, we repeated the analysis four times using different samples from the posterior distribution, with *u*_ext_ = 0. In the chart, we show the number of subject for which at least one sample of parameters produced multistability.

The example in Fig. 5 shows a noisy dynamics around a single fixed point for the two example regions. A detailed investigation reveals that this is a universal trait for all nodes. We consider first the discovered neural mass with all parameters **θ***^r^*, as well as the network input *u* and external input *u*_ext_ set to zero (Fig. 6A). Simulating the dynamics from random initial points, we see that in the absence of noise, the trajectories quickly converge to a single fixed point. All three eigenvalues of the system at this fixed point are real and negative; thus, the fixed point is a stable node.

Does the number of fixed points change for different parameter configurations or different input? To answer, we analyzed the discovered system for 680 inferred configurations of the regional parameters **θ***^r^* (68 nodes for 10 of 100 subjects; only subset of subjects was used because of the computational costs) and nine combinations of *u* and *u*_ext_ (both set to −1, 0, and 1). For each of these configurations, we have located the fixed point(s) numerically by applying nonlinear root finding method to 60 random initial points chosen randomly from the interval [−2, 2] in each dimension (see Methods). Furthermore, from each of these initial points, we simulated the system in the absence of noise to assess whether it exhibits unsteady dynamic, whether periodic or chaotic. In no configuration was multistability observed, and when simulated without the system noise, the system always converged to the fixed point, with no unsteady (periodic or chaotic) behavior (Fig. 6B).

Somewhat unexpectedly, this is mostly also the case when evaluating the dynamics of the whole connected network. Analogously to the previous analysis of a single node, we simulate the dynamics of the coupled network of a single subject in the absence of noise when started from random initial points (Fig. 6C). In this setup, the subject structural connectome is used, all nodes have their inferred parameter values, and external input *u*_ext_ is set to zero. For the example subject, all trajectories converge to a single fixed point. All eigenvalues at the fixed point have a negative real part, but unlike for the isolated node, some eigenvalues are complex, indicating decaying oscillatory dynamics.

We analyzed the dynamical regimes of the coupled network for all subjects analogously to single nodes (Fig. 6D). For all 100 subjects in the dataset, we have used their structural connectome and set the parameters to their inferred values. Choosing random initial conditions, we searched for the fixed points numerically with a root-finding method and again simulated the dynamics in the absence of noise to assess the unsteady behavior. For most of the subjects in the dataset, the state space contains a single stable fixed point. The system contains multiple stable fixed points only for 6 of 100 subjects; however, the effect of this multistability on the generated dynamics is not immediately obvious (fig. S12). Similar to that for the nodal dynamics, in none of the subjects was unsteady behavior observed in the absence of system noise.

#### Three region-specific parameters identified

Next, we turn our attention to the region-specific parameters **θ***^r^*. We first ask the question how many of the parameters can be identified using the data. Luckily, the used framework of variational autoencoders provides a way to answer this question via the so-called posterior collapse (*24*, *25*). The term refers to a phenomenon when the generative model learns to ignore some or all of the latent variables, and the approximate posterior closely matches the prior distribution along these ignored dimensions. In our case, this means that the approximate posterior of the regional parameters *q*(**θ***^r^*∣**y**, **u**, **c**) would collapse to the prior distribution *N*(0, 1) along some or all dimensions. An example of this behavior can be seen on fig. S10.

This phenomenon is sometimes viewed as undesirable, particularly in the extreme case of full collapse along all latent dimensions, as it prevents the use of the latent space leading to suboptimal reconstruction. However, a partial collapse for only a subset of latent variables can be also viewed as a desirable and necessary behavior (*25*), indicating the true dimensionality of data; this is the interpretation that we adopt in this work. Specifically, we expect that as the dimensionality of the parameter space *m_r_* is increased, the necessary dimensionality of the parameter space will be reached, and further dimensions of the parameter space will go unused. This is what we see when we train several models with increasing parameter space dimensionality *m_r_* up to five dimensions (Table 1). Quantifying the collapse phenomenon with the Kullback-Leibler divergence of the approximate posterior from the prior distribution, we see that no more than three dimensions of the parameter space are used even as *m_r_* is increased.

**Table 1.** Quantification of the posterior collapse phenomenon for the regional variables θ*^r^* as the dimensionality of the parameter space is increased. Each column corresponds to a different model with varying numbers of regional parameters *m_r_*; each cell shows the mean and the variance of Kullback-Leibler divergence between the approximate posterior and the prior, $\mathrm{KL}[q(\theta_{j}^{r}|y,u,c)\;\|\;N(0,1)]$, for all regions and all subjects. Because the inference process does not guarantee any specific order of parameter space dimensions, we order each column by the decreasing KL divergence. Values close to zero indicate that the approximate posterior matches the prior distribution. As the dimensionality is increased above *m_r_* = 3, the KL divergences in the first three dimensions remain stable, while the values close to zero in the additional dimensions indicate that the additional dimensions are effectively unused.

*m_r_*	1	2	3	4	5
$\theta_{1}^{r}$	3.313 ± 0.268	2.946 ± 0.209	2.846 ± 0.135	2.744 ± 0.230	2.716 ± 0.133
$\theta_{2}^{r}$	–	2.266 ± 0.935	2.025 ± 0.786	2.238 ± 0.754	2.048 ± 0.714
$\theta_{3}^{r}$	–	–	0.917 ± 0.562	0.713 ± 0.601	0.881 ± 0.515
$\theta_{4}^{r}$	–	–	–	0.002 ± 0.002	0.004 ± 0.007
$\theta_{5}^{r}$	–	–	–	–	0.001 ± 0.000

**Fig. 7.**
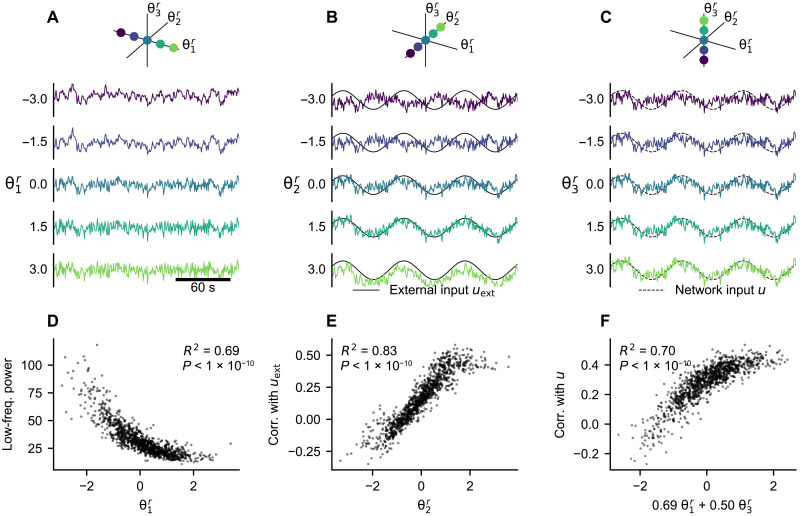
Effect of the regional parameters on the generated data. (**A** to **C**) With the trained model, we simulate the time series of a single region while varying one regional parameter. In each panel, we systematically vary one of the region-specific parameters while keeping others set to zero. The system and observation noise are generated randomly from standard normal distributions but kept the same for all simulations. In (A), the external input and network input are set to zero. In (B), the external input is set to a sine wave with frequency of 0.014 Hz and the network input is set to zero. In (C), the external input is set to zero and the network input is set to a sine wave with frequency of 0.014 Hz. (**D** to **F**) Statistical analysis of the parameter effects illustrated in (A) to (C). We repeatedly (*n* = 1000) simulated the dynamics of a single region with randomly selected **θ***^r^* (see main text). (D) Relation of the low-frequency power (below 0.1 Hz) and $\theta_{1}^{r}$. (E) Relation of the Pearson correlation coefficient of the generated time series with the network input *u*_ext_ and $\theta_{2}^{r}$. (F) Relation of the Pearson correlation coefficient of the generated time series with the network input *u* and the linear combination of $\theta_{1}^{r}$ and $\theta_{3}^{r}$ identified by a multivariate regression (fig. S13).

What is the role of the parameters in the inferred model? We demonstrate their function by generating new data with the trained model, varying a single parameter while keeping others constant and using the same noise instantiation (Fig. 7, A to C). This exploration indicates that the first parameter $\theta_{1}^{r}$ influences the presence of low-frequency (below 0.1 Hz) oscillations. The second parameter $\theta_{2}^{r}$ seemingly modulates the response to the external input: The simulated time series change from being anticorrelated with the external input for negative values to correlated for positive values. Last, the third parameter $\theta_{3}^{r}$ changes the response to the input from the rest of the network, from noncorrelated for negative values to correlated for positive values.

These claims are supported by the statistical analysis of generated time series (Fig. 7, D to F, and fig. S13). We repeatedly simulated the regional dynamics using the trained model and random combination of the parameters **θ***^r^* and evaluated the hypothesised relations. For each simulation (*n* = 1000), the parameters **θ***^r^* were randomly chosen from the prior distribution *N*(**0**, **I**). The system and observation noise were generated randomly and uniquely for each simulation. The external input *u*_ext_ was also generated randomly using the learned parameters. Each region was simulated independently from the others, and the network input was set to be the same for all simulations: The network time series *u* were precomputed using the empirical fMRI data, and we chose to use the time series whose power was at 80 percentile of all data to simulate the nodal dynamics with non-negligible and realistic network input. This analysis shows a clear relation between $\theta_{1}^{r}$ and the low-frequency power of the generated time series, as well as between $\theta_{2}^{r}$ and the correlation of the generated time series with the external input *u*_ext_. The correlation of the generated time series with the network input depends on both $\theta_{1}^{r}$ and $\theta_{3}^{r}$, showing that not every effect is isolated in a single dimension of the parameter space.

Further insight into these can be obtained by the analysis of the inferred dynamical system *f* directly (fig. S14): Analysis of the eigenvalues at the fixed point shows the clear relation of $\theta_{1}^{r}$ with the largest eigenvalue and, thus, the decay time constant. The dependence of the partial derivatives *∂f_i_*/*∂u*_ext_ and *∂f_i_*/*∂u* on parameters **θ***^r^* explains the modulation of the response of the system to external and network input.

Another way of analyzing the roles of region-specific parameter is to look at their relations with various features of the structural and functional data. We divide the features into two categories: First, those derived from the individual data that were used for the model training and, second, those obtained from external sources and not specific to the subject in question. Taking the features and using the inferred parameters for all subjects, we performed a multivariate linear regression for the different features (Table 2). The features considered in this analysis were selected partly on the basis of the understanding gained from the previous analysis and the features of interest in the related studies (*4*–*6*).

**Table 2.** Results of the multivariate linear regression between the means of the inferred regional parameters θ*^r^* and regional features on the individual or population level. Shown are the coefficient of determination *R*^2^ and the regression weights. For visual orientation, the values where *R*^2^ > 0.3 and the absolute value of weight >0.2 are highlighted with an asterisk (*). The weights with *P* > 2.22 × 10^−5^ are marked with a dagger (†) (two-sided *t* test, threshold of 0.001 corrected for 45 comparisons with Bonferroni method). The individual features are calculated from the structural connectivity (SC) or from the processed and parcellated fMRI. External data include average neuronal size and neuronal density, principal gradient of resting state FC, T1w/T2w ratio, first principal component of the gene expression spatial map, and EI map. The analysis was performed using *n* = 6800 data points (100 subjects, 68 regions each). Visualization of the data can be found on figs. S15 and S16. RSFC, resting-state FC.

	Feature	*R* ^2^	Weights		
			$\theta_{1}^{r}$	$\theta_{2}^{r}$	$\theta_{3}^{r}$
Individual data	SC: Node in-strength	0.55*	−0.60*	0.03†	−0.57*
	SC: Node centrality	0.35*	−0.41*	0.17	−0.48*
	fMRI: First PCA eigenvector	0.44*	−0.00†	0.86*	0.05
	fMRI: Second PCA eigenvector	0.01	0.05	0.09	0.04†
	fMRI: Correlation with mean signal	0.58*	0.10	1.00*	0.15
	fMRI: Correlation with network input	0.52*	−0.43*	0.62*	0.51*
	fMRI: Number of zero-crossings	0.96*	0.99*	0.02	−0.01
	fMRI: Power below 0.1 Hz	0.93*	−0.97*	−0.04	0.04
External data	Neuronal size (Von Economo)	0.17	0.38	0.20	−0.14
	Neuronal density (Von Economo)	0.25	−0.46	−0.22	0.20
	Neuronal density (BigBrain)	0.19	−0.42	−0.24	0.02†
	RSFC principal gradient	0.07	0.27	0.07	0.03†
	T1w/T2w ratio	0.19	−0.40	−0.09	0.22
	Gene expression map (first PC)	0.47*	−0.69*	−0.15	0.11
	EI map	0.14	−0.33	−0.23	−0.18

With the individual data, we evaluated the link to features of the structural connectome and the regional fMRI time series. The results correspond well with the effects of the parameters as established above. The first parameter $\theta_{1}^{r}$ is strongly linked with the frequency features of the regional fMRI time series, specifically, the power below 0.1 Hz and number of zero-crossing. The second parameter $\theta_{2}^{r}$ is most importantly linked with the mean fMRI signal and with the first eigenvector obtained from principal components analysis (PCA) of the subject fMRI data. That is consistent with the interpretation that the first principal component corresponds to the external input, the response to which $\theta_{2}^{r}$ modulates. The third parameter $\theta_{3}^{r}$ is linked not only to the features of the structural network, mainly node in-strength, but also to the correlation of the regional fMRI time series with the network input to the same region; this network input also depends on the structural network (Eq. 7). This is also consistent with the modulating effect of $\theta_{3}^{r}$ as established before. The relations between the inferred parameters and the individual data features are visualized on fig. S15.

With the external data, we compared our inferred parameters against multiple feature maps used in previous studies on regional heterogeneity in whole-brain networks (*5*, *6*): neuronal size and density by Von Economo and Koskinas (*26*), neuronal density obtained from BigBrain (*27*), principal gradient of resting-state FC obtained through diffusion embedding (*28*), T1w/T2w ratio approximating the myelin content obtained from HCP cohort data (*29*), the dominant component of brain-related gene expressions (*5*), and the excitation-inhibition (EI) map (*5*) obtained from the Allen Human Brain Atlas (*30*). Only the first of our inferred parameters is strongly linked to any of them, most importantly to the first component of the gene expression spatial map and, to a lesser degree, to the neuronal density from both sources, and to T1w/T2w ratio. The relations between the inferred parameters and the individual data features are visualized on fig. S16.

#### Role of the network coupling in the trained model

**Fig. 8.**
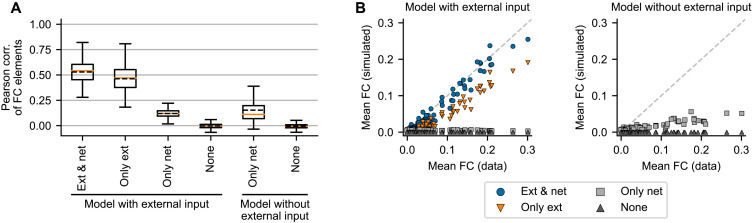
The role of the network connectivity and external input in reproducing the correlation structure. For all 100 subjects in the dataset, we generate new data using the learned regional parameters **θ***^r^*, but with randomly drawn system and observation noise. We use two models: one trained with the external input and one trained without it. We then generate new data with both external input and network input present (Ext & net), with only the external input present and network connectivity set to zero (Only ext), with only the network input and external input set to zero (Only net), and with neither (None). For each subject, we perform 20 simulations with different noise instantiations. (**A**) Pearson correlation of the nondiagonal elements of the FC in the original and generated data for the different variants of the models. Each boxplot is constructed from *n* = 2000 data points. The box extends from the first quartile to the third quartile of the data, with a solid line at the median and dashed line at the mean. The whiskers extend from the box by 1.5× the interquartile range. Fliers are not shown. The differences between all means are statistically significant (two-sided *t* test, *P* < 1 × 10^−10^) except between the two variants without either input. (**B**) Mean of the nondiagonal elements of the original and simulated FC. Each dot corresponds to the single subject; the FC mean was averaged over the 20 simulations.

Figure 5 illustrates that the trained model can generate network dynamics with correlation structure resembling the original data. Evaluated for all subjects, the trained model produces dynamics that matches the FC of the original data with the mean Pearson correlation coefficient 0.53 (Fig. 8A, leftmost column). While this is lower than what was obtained in related studies (we will return to this fact in Discussion) (*3*–*6*), it shows that a considerable portion of functional network structure is captured by the model. We however note that FC dynamics (FCD), that is, time-varying changes in the FC, does not match the data (fig. S18).

Considering these results and the presence of the external input, we can ask what role does the structural connections between the brain regions play in the model and to what extent is the correlation structure caused by the external input. We investigate this by comparing how well the structure is reproduced with several variations of the model (Fig. 8). We consider the trained model and generate new data, assuming that the external input and network input are either present or artificially set to zero. We also consider the model that is trained without the external input in the first place.

The results indicate that the generated correlation structure is mainly caused by the external input, as evidenced by the drop of FC similarity when the external input is absent, irrespective of whether the model was trained with or without it. The same applies for the overall strength of the simulated FC. Nevertheless, the role of the network connectivity is not negligible, and it improves the performance. Only a minor improvement is achieved if the model is trained without the external input in the first place, and thus, we cannot rely on it for the correlation structure.

In the analysis performed up to now, we have used the structural connectome as estimated from diffusion imaging tractography via streamline counting. In studies focused on large-scale brain dynamics, several variants of the structural connectome preprocessing were proposed; these modifications can increase the fit of the simulated and original FC (*31*). How large of an influence do these preprocessing choices have here? We compare four preprocessing variants (with or without log scaling and with or without added homotopic connections; fig. S17) and conclude that it is relatively minor. The differences when using the model with external input are small (mean FC correlation between 0.529 and 0.560 for all variants), likely because the external input dominates. More interesting differences can be seen for the model without the external input; we particularly note the positive effect of strengthening the homotopic connections (mean FC correlation of 0.178 to 0.237 for linear scaling and 0.177 to 0.211 for log scaling).

## DISCUSSION

### Main results

The main contribution of this work is a method for analysis of whole-brain dynamics based on a data-driven model of networked dynamical systems. Using the structure of the network and the functional data, the method allows us to infer both the unknown generative dynamical system and the parameters varying across regions and subjects. It allows us to analyze the large-scale brain dynamics and the regional heterogeneity without strong assumptions on the underlying dynamical system and thus provides a way for model-based analysis of brain dynamics.

We have tested the method on two synthetic datasets, one generated by a network model composed of nodes with a Hopf model (Fig. 2) and one generated by a network model with a pMFM (Fig. 3). Detailed analysis of the results has shown that the proposed method can recover the original parameters and reproduce the important features of the original data both on the single-region level and on the network level (Fig. 4). However, the strength of the FC was underestimated for both test cases.

We then applied the method on human resting-state fMRI for 100 subjects from HCP. This application and the detailed analysis led to several interesting results. First, the discovered dynamics both on the node level and on the network level can be mainly characterized as noise-driven fluctuations around a single fixed point (Fig. 6). Second, the method was able to reliably recover three, and only three, regional parameters with clear and distinct effects on the generated dynamics (Fig. 7), one of which is strongly correlated with the first principal component of the gene expression spatial map (Table 2). Third, the learned model was able to partially reproduce the correlation structure of the original functional data, although to a lesser extent than what was achieved in previous works. To do this, it strongly relied on the external input and only weakly on the network connectivity (Fig. 8). We discuss these results in the following paragraphs.

### Discovered dynamics on the node and network level

Applied to the resting-state fMRI data, our trained model was able to reproduce the correlation structure of the original data to a large degree. Unlike many previous computational studies linking the structural and the FC (*2*), our data-driven model relied strongly on the external input to explain the correlation structure, and the network coupling played only a secondary role (Fig. 8). Furthermore, the trained model was not able to produce realistic time-dependent variations of the FC. The discovered dynamical regime of a single node level can be characterized as noisy fluctuations around a single stable fixed point. The nodes are weakly coupled together in the network, leading to noisy dynamics around a single fixed point also on the network level for most of the subjects. This inferred dynamical system mostly resembles the Hopf bifurcation network model (*8*), with all nodes in the subcritical regime and with weak network coupling but with the added external input that compensates for the weak coupling and forces the desired correlation structure.

Our results, to some extent, conflict with the previous literature, which demonstrated that a notable better match of FC matrices and better similarity of FCD can be obtained with heterogeneous network-based models. Moreover, previous models achieved the better fit without the use of external input (*3*–*6*). Furthermore, the model fitting of such network-based models often indicated that the local dynamics relies on nonlinear phenomena, such as stable oscillatory dynamics, multistability, and multiple distinct dynamical regimes in the plausible parameter range. While it was demonstrated that good match of static FC can be achieved even with relatively simple models with single fixed-point dynamics (*32*), nonlinear dynamics is crucial in generating rich temporal behavior matching that of the observed data (*33*). Considering that our method was able to discover only a single fixed-point dynamics, it is thus expected that the model produced only unsatisfactory match of the time-varying FC. Together, we view these aspects of our results as a reflection of possible shortcomings of the inference algorithm rather than a strong statement on the nature of brain dynamics. We consider several factors that might have contributed to these results: the optimization target, the used method for system identification, fMRI preprocessing, fixed structural connectivity, and assumption on the coupling via the observed variable.

#### Optimization target

First, let us comment on the methodological differences between previous network-based modeling studies and system identification approach that we have adopted here. Previous works demonstrated that better match of static FC and of FCD can be achieved with network-based models of large-scale brain dynamics (*3*–*6*). These works started with a well-constrained model (in terms of node dynamics and network structure) and tuned the model parameters so a specified feature, such as FC or FCD, was matched as closely as possible. This was achieved through parameter optimization using the similarity of the specified features as the cost function. Notably, the generated time series were not required to resemble the original data in any other aspect than the specified feature.

In contrast, our goal here is to learn the generative model of the observed neural activity, that is, a model that generates data resembling those observed in all aspects, as much as possible given its architectural constraints. Because we are assuming that the studied system is noise driven, there is a limit to this similarity: We should not expect the generated time series to match the training data any more than a new observation of the system would. Thus, the exact time series might differ, but ideally, the features that would be (at least partially) preserved in a new observation, such as FC, time-independent features of FCD, or energy and frequency spectra of individual time series, should be preserved in the simulated signals too, to the same extent as with the repeated observation.

We achieve this objective using the variational autoencoder framework. Under this framework, the cost function is composed of the reconstruction quality term (Eq. 13), which quantifies the similarity of the time series when the system states are drawn from a posterior distribution and of the posterior regularization terms (Eqs. 14 and 15). If the architecture is sufficiently expressive and can be well trained, then the aggregated posterior distribution [$q^{*}(x)=\frac{1}{N}\sum_{i=1}^{N}q(x|y_{i},u_{i},c_{i})$], with *i* indexing over all regions and subjects) (*34*) covers the prior distribution due to the influence of the regularization term. This, in turn, means that a simulation with noise drawn from the prior distribution will produce time series resembling those in the training data.

To summarize, with our approach, the match of the observed and simulated FC is desirable and can be used as a measure of the quality of the trained model, but because it is not directly optimized, it is of little surprise that other methods that target it directly can achieve better results. What we are getting instead is a generative model that strives to mimic the original data in all aspects, without specifying which data features should be targeted.

#### Method of system identification

Undoubtedly, the choice of the parameterization of the function *f* in the dynamical system and the learning algorithm used affects the nature of the dynamics that can be and is learned from the data. Our method led to the discovery of a system that mainly relied on a single fixed point dynamics, of which one consequence is that the trained system was not able to reproduce the complex time-varying FC of the resting-state fMRI. Therefore, a desirable goal for the future studies is a stronger inference method that can overcome this shortcoming. Such goal requires that the parameterization of function *f* is sufficiently expressive that it can well represent a wide range of nonlinear dynamics, and at the same time, the optimal parameter configuration is reachable with the chosen optimization algorithm. Specifically for our problem, this has to hold when the neural mass model to be learned is embedded in the network; we return to this coupling issue in the later paragraph.

Fortunately, existing work offers many variations of system identification methods, and although they were not applied in a networked setting, that can be explored when searching for a method with improved convergence properties: For instance, Duncker *et al.* (*11*) relied on Gaussian processes conditioned on a set of fixed points to learn the system dynamics and demonstrated its efficacy on multistable dynamical systems. Nassar *et al.* (*13*) used a tree structure to partition the state space and approximate the system in each partition with linear dynamics. Koppe *et al.* (*16*) used piecewise linear recurrent neural network to analyze fMRI data. Schmidt *et al.* (*14*) later expanded on this work, introducing an approach for better approximation of systems with multiple time scales through the creation of slow manifolds in the state space using a regularization scheme of the dynamical system.

It is worth emphasizing that it is not just the parameterization of the function *f* that needs to be considered but the whole training framework: In an extensive benchmark of their architecture for dynamical system identification, Brenner *et al.* (*35*) have shown notable performance differences for the same architecture trained as a deterministic system by backpropagation with teacher forcing or a stochastic system trained by variational inference method.

#### fMRI preprocessing

It is well established that fMRI data contain a variety of structured spatial and temporal artifacts of non-neuronal origins. It is however debated how to distinguish and remove the widespread deflections of non-neuronal origin (which are implied to be irrelevant for understanding the brain dynamics) while retaining the spatially structured signal caused by the neuronal sources (*23*, *36*, *37*). In the context of large-scale brain modeling, leaving the widespread signal deflections can lead to fitting irrelevant artifacts. On the other hand, removing them with methods such as global mean regression risks removing relevant information and introducing artificial anticorrelations.

An illustrative example of the dangers of preprocessing was recently provided by Aquino and colleagues (*38*). In their study, the authors examined three approaches toward removing widespread deflection in the fMRI data: without correction, using the DiCER method (*23*) for diffuse cluster estimation and regression, and with global mean regression. They demonstrated that the results of the model fitting, that is, the estimated model parameters, depend strongly on the chosen method for fMRI preprocessing.

In this study, we have used the DiCER method (*23*), designed for a removal of widespread deflections, which is more fine-grained than global mean regression. Still, considerable widespread fluctuations remained, which explains the strong role of external input in our trained model and why the model was able to match the FC relatively well even without the network connectivity. For future studies, we urge that the effects of the fMRI preprocessing are carefully considered and tested. A flexible way toward dealing with the issue can be, instead of preprocessing, integrating the suspected neuronal and non-neuronal sources into the model directly. This way, the relevant and irrelevant sources of signal can be investigated in one framework; however, the more complex model would make the model fitting even more challenging.

#### Fixed structural connectivity

Our method assumes that the structural connectome through which the local dynamics is coupled is known. What we can obtain, however, is only an estimate from diffusion tractography, suffering from a range of biases (*39*, *40*). Our results indicate that while the method can handle small perturbations of the connectome, larger perturbations or different scaling can considerably degrade its performance (fig. S9). Conversely, correcting the structural connectome for known biases can improve the model fit, as demonstrated by the better performance of the model on HCP data when homotopic connections are strengthened (fig. S17).

Further handmade corrections to the structural connections can be considered, but an alternate approach can be also pursued: one that would use the estimated structural connectome not as hard data but only as a soft prior for the effective connectivity of the model. Such approach was described for whole-brain dynamics generated by the multivariate Ornstein-Uhlenbeck process, using the thresholded structural connectivity as a topological mask for the inferred effective connectivity (*41*, *42*). The model connectivity may be inferred even without any prior anatomical constraints, as demonstrated by the Mesoscale Individualized Neurodynamics (MINDy) method that relies on a simple one-equation neural mass model (*17*).

#### Coupling via the observed variable

One of the more debatable choices that we have made in designing our method is the assumption that the regions are coupled via the observed variable, that is, that *g_c_* ≡ *g* in Eqs. 4 to 6. This assumption was motivated purely by pragmatical reasons, as it allows us to decouple the networked problem: With the assumption, the network input of each region can be calculated beforehand and stays fixed during the optimization. Effectively, this means that we are performing the inference for *n* uncoupled regions instead of one network with *n* nodes, reducing the computational resources needed.

We justify the choice by two simple facts: (i) It leads to a problem that can be efficiently solved, and (ii) the designed method is shown to perform reasonably well on the synthetic test cases, even if the assumption is clearly invalid. For instance, in the original Hopf model, the regions are coupled via both variables, yet the trained model produces dynamics with similar correlation structure. At the same time, we note that the network coupling was reduced in both synthetic test cases, and this assumption is a plausible explanation of this reduction. Similarly, it might have been part of the reason why we have not been able to reproduce the FC with the human fMRI data to the same degree as similar computational studies. The design of a computationally efficient method for system identification of fully coupled systems is thus an important direction for future studies.

#### Related works on system identification from fMRI signals

Diversity of the results on the dynamical nature of large-scale fMRI signals highlights the importance of the modeling choices, particularly that of the underlying dynamical model and the optimization cost function. Nozari and colleagues (*43*) compared several classes of dynamical models for their capacity to model whole-brain resting-state fMRI dynamics, from linear models to nonlinear neural mass models to deep neural network models. They conclude that the best performance is achieved by linear autoregressive models, and more complex models lead to reduction in performance and increase in computational costs. However, it is worth noting that the models are trained for and evaluated on the one-step prediction capacity, and it is unclear how well the tested models would perform with other criteria such as the static or time-varying FC or frequency spectra.

Piccinini and colleagues (*44*) used the framework of network-based model of whole-brain dynamics combined with SINDy-like approach (*9*). They represented the 2D neural mass model with flexible polynomial form and optimized its coefficients, targeting the best match of simulated and empirical FC. Their best models exhibited a range of local dynamics, most of them having a single fixed point with either a stable fixed point or a limit cycle, but configurations with three or five fixed points were also common. From the reported results, it is however difficult to establish how well the models match the original data in other aspects than the evaluated FC criteria.

It is also illuminating to compare our results with those of Koppe and colleagues (*16*), who, despite using a conceptually similar methodology of system identification from fMRI time series, arrived at considerably different results regarding the nature of the underlying dynamical system. Using piecewise-linear recurrent neural networks, the authors reconstructed dynamical systems generating the fMRI time series. They investigated task-based fMRI and analyzed each region of interest independently from the others. They discovered rich nonlinear dynamics in the inferred systems, including limit cycles on several time scales, multistability, or chaotic attractors, a stark contrast to our single fixed point systems.

We speculate that there are three principal reasons behind this difference. First is the source of the data itself: task-based fMRI compared to our resting-state fMRI. Unlike resting-state fMRI, the task setting introduces changes in the brain dynamics on the task time scale, which we presume are then reflected in the organization of the state space and the dynamical structures on the task time scale. The second reason is the used fMRI preprocessing: Unlike us, Koppe and colleagues (*16*) smoothened the fMRI signal used for the system identification. It is seemingly a minor difference yet introduces predictability on the short time scales of the smoothing window, which might lead to preference for explanation via complex deterministic dynamics rather than noisy fluctuations. Put differently, not smoothing the time series leave our method with the need to model one additional source of noise, and because that can be done also on the level of system noise, this preprocessing choice might lead to widening of the basis of attraction of the noisy fixed-point dynamics in the cost function landscape, leaving the more complex dynamical regimes harder to discover. Last, the differences may be caused by subtle priors (intentional or not) of the system identification method. Consider sufficiently complex observed dynamics, fitted by a model of a high-dimensional deterministic system and a low-dimensional stochastic system. While the first one might try to explain the observed dynamics with complex chaotic dynamics, the second might rely on the noisy dynamics, having nothing else at its disposal. A model of high-dimensional stochastic system, however, might fit the data with either the deterministic chaotic dynamics or stochastic noise, or anything in between, depending on the intentional and unintentional priors of the method. What matters, ultimately, is the quality of the reconstruction of the original signal, requiring careful benchmarks of the inferred models.

#### Cortical gradients in large-scale brain modeling

Gradients of structure, connectivity, gene expression, and function across the human cortex raised considerable interest in recent years (*45*, *46*), and several modeling studies investigated their role in the large-scale brain dynamics (*3*–*6*). Our approach shares with them the basic principles of large-scale brain modeling based on the structural connectome but differs in three key points: First, we do not rely on any particular neural mass model; rather, the model is derived from data. Second, the regional parameter that we infer are not constrained by any particular parameterization. Third, via the cost function, we are fitting the time series directly and not any derived feature such as static or dynamical FC.

How do our results compare to those of previous studies in terms of the regional heterogeneity of the parameters? Demirtaş *et al.* (*3*) used a two-population neural mass model embedded in the whole-brain network. They parameterized the neural mass parameters with the T1w/T2w ratio approximating the myelin content and optimized the model parameter to obtain best FC fit. They found that the heterogeneity following the T1w/T2w gradient improves the fit compared to the homogeneous model or random surrogates. Wang *et al.* (*6*) used a one-equation neural mass model in the network, whose parameters were optimized freely to, again, fit the FC matrix. They found that the optimal parameters are correlated with the first principal gradient of resting-state FC (RSFC) and with the T1w/T2w map. Furthermore, the optimal strength of recurrent connection was correlated with the neuronal density map. Expanding the work, Kong *et al.* (*4*) parameterized the same model with the T1w/T2w map and RSFC gradient to fit not only the static but also the dynamical FC. For this goal, parameterization with both maps was necessary. These conclusions are in general agreement with our results: The first inferred parameter is correlated with the T1w/T2w map, RSFC gradient, and neuronal density, although the relation is not as strong, particularly for the RSFC gradient (*R*^2^ = 0.07 in our case).

Kong *et al.* (*4*) however also noted that their optimal regional parameters are strongly linked with the gene expression gradients, particularly with the first principal component of expression maps of 2413 brain-specific genes. This is a relation that our study strongly supports, as the first inferred parameter was strongly linked to the first principal component of the gene expression spatial map (*R*^2^ = 0.47). In addition, Deco *et al.* (*5*) investigated the role of gene expression in whole-brain dynamics. They compared the goodness of fit when the neural mass model was parameterized with EI ratio (E:I) obtained from gene expression, as well as with first principal component of gene expression and T1w/T2w ratio or using a homogeneous model. The fit was best in the E:I case, followed by the other heterogeneous models and then the homogeneous one. This result is not supported by this study, as the EI map was correlated to the inferred parameters only weakly.

We have chosen to perform the analysis using the Desikan-Killiany cortical parcellation (*20*), mainly for consistency with related studies (*4*–*6*). It is well recognized that the choice of brain atlas can affect the results of neuroscientific studies (*47*). Future applications of the proposed framework might thus benefit from analyses on not only anatomy-based parcellations such as Desikan-Killiany but also multimodal (*29*) or cytoarchitectonic (*48*) parcellations, which should better reflect the underlying structural organization.

#### Inferring subject-specific parameters

The application of our proposed method on the resting-state fMRI data revealed convergence issues for the subject-specific parameters (fig. S10); for this reason, we have used only the region-specific parameters in the main analysis. The precise reason of this failure is not fully clear; however, there are several options that might improve the behavior in future works.

In the framework of variational autoencoders (VAE), one option would be increasing the Kullback-Leibler divergence penalty between the prior and the approximate posterior in the cost function following the so-called β-VAE approach. Proposed for the unsupervised discovery of disentangled representations, β-VAEs (*49*) constrain the capacity of the latent space via an adjustable hyperparameter β and encourage tighter adherence to the prior at the expense of reconstruction quality. Since its proposal, the performance of the approach and the precise definition of disentanglement have been questioned (*50*, *51*). In our case, however, it provides a straightforward way toward better-behaved approximate posterior distributions, although at the expense of an additional parameter that needs to be tuned heuristically.

Another option arises from the conjecture that the failure is caused by imposing a particular structure of the latent space in the generative model, which is not appropriately reflected in the inference model. Here, the inspiration can be taken from the literature on hierarchical VAEs (*52*–*54*). In these works, a particular structure of the latent space is introduced by partitioning the latent space **z** = {**z**_1_, …, **z***_n_*} and factorizing the approximate posterior for data **x** as *q*(**z**∣**x**) = ∏*_i_q*(**z***_i_*∣**z**_<*i*_, **x**), leading to increased flexibility of the model. Modification of our inference model along similar lines could rectify the observed issues.

#### Outlook on the role of dynamically relevant parameters

Large-scale brain dynamics during resting-state is altered in neurodegenerative diseases (*55*) and in normal aging (*56*). Myriads of regionally varying parameters that can plausibly influence the large-scale dynamics can be measured either in vivo or post mortem, such as cell density, cell type composition, local connectivity structure, connectivity to subcortical structures, or receptor densities, to name just a few. But which ones are in fact relevant for large-scale brain dynamics, and how do they influence it? Construction of bottom-up mechanistic models that would include all possible parameters and allow us to investigate their role is unfeasible because of the complexity of human brain with its dynamics spanning multiple temporal and spatial scales, even if the parameters were accurately measured (*57*).

Our approach instead pursues this understanding from the opposite direction. We use the amortized inference framework to learn the dynamical system driving the dynamics and, with it, also the parameters varying across regions and subjects. Because these parameters are inferred from the functional data in an unsupervised fashion, they are, by construction, the parameters relevant for the large-scale dynamics. That is in contrast to mechanistic models, whose parameters. while present in the model formulation, might not necessarily affect the observed large-scale dynamics and are thus nonidentifiable from the functional data. Given the abstract nature of the inferred model, the mechanistic meaning of these dynamically relevant parameters is not self-evident, yet they still provide a measure of similarity of brain regions and different subjects, and their effect on the dynamics can be investigated through the trained model. Furthermore, given a large enough dataset, the dynamically relevant parameters may be linked to the measured quantities (or their combinations). Such link may provide insights into the origin of neurodegenerative diseases if the dynamically relevant parameters differ between the disease stages.

The link between dynamically relevant parameters and the measurable quantities can be estimated from a preexisting patient cohort and then only applied to a single subject. That is advantageous if the measurement is difficult, costly, or impossible to perform in clinical setting (such as for cell type composition estimated from post mortem studies); in such cases, the dynamically relevant parameters may instead be estimated from easy-to-obtain resting-state fMRI and then mapped using the known link. This approach thus opens new possibilities for exploitation of large-scale neuroimaging databases such as HCP (*19*) or UK Biobank (*58*) on one hand and detailed cytoarchitectonic (*27*, *48*) or genetic (*30*) brain atlases on the other.

## METHODS

### Structural connectomes

The structural connectomes used for the application on empirical data, as well as for the generation of synthetic datasets, were derived from the neuroimaging data from HCP (*19*). The Washington University–University of Minnesota (WU-Minn HCP) Consortium obtained full informed consent from all participants, and research procedures and ethical guidelines were followed in accordance with Washington University institutional review board approval (Mapping the Human Connectome: Structure, Function and Heritability; IRB #201204036).

Specifically, we used the 100 Unrelated Subjects group from the HCP 1200 Subjects cohort. For those, Structural Preprocessed and Diffusion Preprocessed packages were downloaded (*59*). Next, the structural connectomes were built for the cortical regions of Desikan-Killiany parcellation (*20*) using MRtrix 3.0 (*60*). To do so, first the response function for spherical deconvolution was estimated using the dhollander algorithm (*61*). Next, fiber orientation distribution was estimated using multi-shell, multi-tissue constrained spherical deconvolution (*62*). Then, 10 million tracks were generated using the probabilistic iFOD2 (second-order integration over fiber orientation distributions) algorithm (*63*). These were then filtered using the Spherical-deconvolution Informed Filtering of Tractograms (SIFT) algorithm (*64*). Last, the connectome were built by counting the tracks connecting all pairs of brain regions in the parcellation.

Four variants of the structural connectomes were used for the application on empirical data: The standard variant corresponds to the description above. The log-scaled connectome was calculated as $W_{\log}=\log_{10}(W+10^{q})$, with *q* = −3 and *W* being the original connectome. Both the standard and log-scaled connectomes were also modified by strengthening the homotopic connections, that is, those connecting corresponding regions in the opposite hemispheres. For this, the strength of all homotopic connections was set to 97 percentile of all values in the structural connectome. The perturbed connectomes were constructed by taking the original connectome *W* and adding a matrix with elements from random normal distribution, scaled by the perturbation magnitude ϵ, i.e., *W*_ϵ_ = *W* + ϵ*A*. For each value of perturbation magnitude, four different perturbed connectomes were built. In all cases, the connectome matrices were normalized so that the largest element in each was equal to one.

### Resting-state fMRI data

The resting-state fMRI data were obtained from the HCP for the same subjects as the structural data. We have used the resting-state data preprocessed by the HCP functional pipeline and ICA-FIX pipeline (Resting State fMRI FIX-Denoised package). These were further processed by the DiCER method (*23*) with default parameters. The DiCER method was designed to remove widespread deflection from the fMRI data, and provide a better alternative to global signal regression. Afterward, the processed data were parcellated into 68 regions of Desikan-Killiany parcellation (*20*), and each time series was normalized to zero mean and unit variance. The DiCER preprocessing was performed for all four sessions (REST1_LR, REST1_RL, REST2_LR, and REST2_RL) concatenated. However, only the results from the first session (REST1_LR) were used in the study. The time series were thus 14.4 min long (1200 samples with 0.72-Hz sampling frequency).

### Amortized variational inference for networks of nonlinear dynamical systems

#### Generative dynamical system

As outlined above, we assume that the observed activity *y_j_*(*t*) of a brain region *j* is generated by a dynamical system

$${\dot{x}}_{j}(t)=f[x_{j}(t),{\text{θ}}_{j}^{r},{\text{θ}}^{s},u_{\mathrm{ext}}(t),u_{j}(t)]+{\text{η}}_{j}(t)$$ (4)

$$y_{j}(t)=g[x_{j}(t)]+\nu_{j}(t)$$ (5)

where **x***_j_*(*t*) ∈ ℝ*^n_s_^* is the state at time *t*, ${\text{θ}}_{j}^{r}\in R^{m_{r}}$ and **θ***^s^* ∈ ℝ*^m_s_^* are the region-specific and subject-specific parameters, and *u*_ext_(*t*) is the external input, shared by all regions of a single subject

$$u_{j}(t)=\sum_{i=1}^{n}w_{ji}g_{c}[x_{j}(t)]$$ (6)

is the network input to region *j* with ${\left\{w_{ij}\right\}}_{i,j=1}^{n}$ being the structural connectome matrix of the network with *n* nodes.

To make the inference problem more tractable, we simplify the problem and assume that the nodes are coupled through the observed variable *y_j_*. More precisely, we assume that in Eqs. 4 to 6, *g* ≡ *g_c_* and that the observation noise term *ν_j_* is small enough that it can be included in the coupling. Then, the network input has the form

$$u_{j}(t)=\sum_{i=1}^{n}w_{ji}y_{i}(t)$$ (7)

This form has the advantage that the network input is independent of any hidden variables and can be computed directly from the known observations *y_j_*. This effectively decouples the time series in different nodes so that they can be processed separately, as described below.

For the purpose of the inference, we use the time-discretized form of Eqs. 4 and 5 using the Euler method

$$x_{j,k+1}=x_{j,k}+{\text{Δ}}_{t}f(x_{j,k},{\text{θ}}_{j}^{r},{\text{θ}}^{s},u_{\mathrm{ext},k},u_{j,k})+{\text{η}}_{j,k}$$ (8)

$$y_{j,k}=g(x_{j,k})+\nu_{j,k}$$ (9)

where we denote the time step with the index *k*.

#### Evidence lower bound

As usual in variational inference, we aim to maximize the ELBO and, by doing so at the same time, minimize the Kullback-Leibler divergence between the true posterior and the approximate posterior *q*. In the following text, we consider only a single data point from one subject and one region and omit the region indexing for brevity.

A single data point {**y**, **u**, **c**} representing the data from a one region is composed of the observed time series **y** ∈ ℝ*^n_t_^*, network input time series **u** ∈ ℝ*^n_t_^*, and one-hot vector **c** ∈ ℝ^*n*_sub_^, that is, a vector with zeros everywhere except *i*-th position with value one, encoding the identity of subject *i*. For this data point, the ELBO can be expressed as follows. (For details see the Supplementary Materials.)

$$\;L=E_{q}[\log\;p(y|x,{\text{θ}}^{r},{\text{θ}}^{s},u_{\mathrm{ext}},u)]$$ (10)

$$+E_{q}[\log\;p(x|{\text{θ}}^{r},{\text{θ}}^{s},u_{\mathrm{ext}},u)]+E_{q}[\log\;p({\text{θ}}^{r})]+\frac{1}{n}E_{q}[\log\;p({\text{θ}}^{s})]+\frac{1}{n}E_{q}[\log\;p(u_{\mathrm{ext}})]$$ (11)

$$\;-E_{q}[\log\;q(x|y,u,c)]-E_{q}[\log\;q({\text{θ}}^{r}|y,u,c)]-\frac{1}{n}E_{q}[\log\;q({\text{θ}}^{s}|c)]-\frac{1}{n}E_{q}[\log\;q(u_{\mathrm{ext}}|c)]$$ (12)

Here, the first line represents the decoder loss; second line represents the priors for states **x**, region- and subject-specific parameters **θ***^r^* and **θ***^s^*, and the external input **u**_ext_, and the third line represents the approximate posteriors again for states, region- and subject-specific parameters, and the external input. Alternatively, the second and third line above can be rewritten using the Kullback-Leibler divergences of the posterior and prior distributions

$$\;L=E_{q}[\log\;p(y|x,{\text{θ}}^{r},{\text{θ}}^{s},u_{\mathrm{ext}},u)]$$ (13)

$$-\mathrm{KL}[q(x|y,u,c)\;\|\;p(x|{\text{θ}}^{r},{\text{θ}}^{s},u_{\mathrm{ext}},u)]-\mathrm{KL}[q({\text{θ}}^{r}|y,u,c)\;\|\;p({\text{θ}}^{r})]$$ (14)

$$\;-\frac{1}{n}\mathrm{KL}[q({\text{θ}}^{s}|c)\;\|\;p({\text{θ}}^{s})]-\frac{1}{n}\mathrm{KL}[q(u_{\mathrm{ext}}|c)\;\|\;p(u_{\mathrm{ext}})]$$ (15)

### Decoder or the observation model

We assume that the observation model can be modeled as a linear transformation of the system state with Gaussian noise, *y* = *g*(**x**) + ν = ***a*** · **x** + *b* + ν. This forward projection essentially represents the decoder part of the encoder-decoder system, so the likelihood in Eq. 10 can be expanded over time as

$$p(y|x,{\text{θ}}^{r},{\text{θ}}^{s},u_{\mathrm{ext}},u)=\prod_{k=1}^{n_{t}}p(y_{k}|x_{k})=\prod_{k=1}^{n_{t}}N(y_{k}|a\cdot x_{k}+b,\sigma_{o}^{2})$$ (16)

where *N*(*y*∣μ, σ^2^) stands for normal distribution with mean μ and variance σ^2^. The parameters of the observation model, which are to be optimized, are the coefficients of the linear projection **a** and *b*, together with the observation noise variance $\sigma_{o}^{2}$.

#### Prior on the system states

The first term in Eq. 11 represents the prior function on the system states **x** given the network input **u**, external input **u**_ext_, and the parameters **θ***^r^* and **θ***^s^*. It is here where the dynamical system *f* appears in the ELBO. This term can be expanded over time as

$$\begin{array}{rl} p(x|{\text{θ}}^{r},{\text{θ}}^{s},u_{\mathrm{ext}},u) & =p(x_{0})\prod_{k=1}^{n_{t}}p(x_{k+1}|x_{k},{\text{θ}}^{r},{\text{θ}}^{s},u_{\mathrm{ext},k},u_{k})\\ & =N(x_{0}|0,I)\prod_{k=1}^{n_{t}}N[x_{k+1}|x_{k}+{\text{Δ}}_{t}f\\ & \;\times (x_{k},{\text{θ}}^{r},{\text{θ}}^{s},u_{\mathrm{ext},k},u_{k}),\mathrm{diag}(\sigma_{s}^{2})] \end{array}$$ (17)

Here, we use the standard normal distribution as a prior for the initial state **x**_0_ and then evolve the system over time according to the function *f*. We represent the function *f* as a two-layer neural network, with a rectified linear unit (ReLU) activation function in the hidden layer. That is, with $\tilde{x}=(x_{k},{\text{θ}}^{r},{\text{θ}}^{s},u_{\mathrm{ext},k},u_{k})\in R^{n_{i}}$ denoting the concatenation of the system state, regional parameters, subject parameters, external input, and network input with size *n_i_* = *n_s_* + *m_r_* + *m_s_* + 2, function *f* : ℝ*^n_i_^* → ℝ*^n_s_^* is given as

$$f(\tilde{x})=W_{2}\phi (W_{1}\tilde{x}+b_{1})+b_{2}$$ (18)

with **W**_1_ ∈ ℝ^*n_h_*×*n_i_*^, **W**_2_ ∈ ℝ^*n_s_*×*n_h_*^, **b**_1_ ∈ ℝ*^n_h_^*, and **b**_2_ ∈ ℝ*^n_s_^* being the weights and biases, *n_h_* being the number of hidden units, and ϕ(**x**) = max (0, **x**) being the ReLU rectifier. The weights and biases of the network are to be optimized, together with the system noise SD **σ***_s_*. The number of hidden units is given in the table S1.

#### Prior on the parameters

For the region- and subject-specific parameters, we use the standard normal distribution as a prior, as is often used in variational autoencoders. The priors in the second and the third term in Eq. 11 can thus be written as *p*(**θ***^r^*) = *N*(**θ***^r^*∣**0**, **I**) and *p*(**θ***^s^*) = *N*(**θ***^s^*∣**0**, **I**).

#### Prior on the external input

We set the prior for the external input to an autoregressive process with time scale τ and variance σ^2^. Then, the prior reads

$$p(u_{\mathrm{ext}})=p(u_{\mathrm{ext},0})\prod_{k=1}^{n_{t}}p(u_{\mathrm{ext},k+1}|u_{\mathrm{ext},k})$$ (19)

$$=N(u_{\mathrm{ext},0}|0,\sigma^{2})\prod_{k=1}^{n_{t}}N(u_{\mathrm{ext},k+1}|\alpha u_{\mathrm{ext},k},\sigma^{2})$$ (20)

with α = *e*^−1/τ^. The variance is fixed to σ = 1 because any scaling can be done inside the function *f*, and the time scale is optimized together with the other parameters and neural network weights in the optimization process.

#### Approximate posteriors

We follow the standard approach and use multivariate normal distributions for the approximate posteriors in Eq. 12. For the states **x** and region-specific parameters **θ***^r^*, we use the idea of amortized variational inference, and instead of representing the parameters directly, we train a recurrent neural network to extract the means and the variances from the time series of the observations **y**, time series of the network input **u**, and the one-hot vector **c** encoding the subject identity

$$\left({\text{μ}}_{x},\log\;{\text{σ}}_{x}^{2})=h_{1}(y,u,c\right)$$ (21)

$$q(x|y,u,c)=N[x|{\text{μ}}_{x},\mathrm{diag}({\text{σ}}_{x}^{2})]$$ (22)

and

$$\left({\text{μ}}_{r},\log\;{\text{σ}}_{r}^{2})=h_{2}(y,u,c\right)$$ (23)

$$q({\text{θ}}^{r}|y,u,c)=N[{\text{θ}}^{r}|{\text{μ}}_{r},\mathrm{diag}({\text{σ}}_{r}^{2})]$$ (24)

Specifically, we use long short-term memory (LSTM) networks for both functions *h*_1_ and *h*_2_. The input to the networks at step *k* is the concatenated observation *y_k_* and the network input *u_k_*, to which the time-independent one-hot vector **c** is also appended.

In other words, the trained LSTM networks map the time series of observations **y**, time series of the network input **u**, and identity vector **c** to the parameters of multivariate normal distributions with diagonal covariance, which define the approximate posterior distributions of the state time series **x** and regional parameters **θ***^r^*.

The subject-specific parameters **θ***^s^* and the external input **u**_ext_ depend only on the subject identity encoded in the one-hot vector **c**. Their means and variances are stored directly in the matrices of means (**M**_1_ ∈ ℝ^*m_s_*×*n*_sub_^ for **θ***^s^* and **M**_2_ ∈ ℝ^*n_t_*×*n*_sub_^ for **u**_ext_) and matrices of log-variances (**V**_1_ ∈ ℝ^*m_s_*×*n*_sub_^ and **V**_2_ ∈ ℝ^*n_t_*×*n*_sub_^). For a specific subject, the relevant values are extracted through the product with the one-hot vector **c**

$$\left({\text{μ}}_{s},\log\;{\text{σ}}_{s}^{2})=h_{3}(c)=(M_{1}\cdot c,V_{1}\cdot c\right)$$ (25)

$$q({\text{θ}}^{s}|c)=N[{\text{θ}}^{s}|{\text{μ}}_{s},\mathrm{diag}({\text{σ}}_{s}^{2})]$$ (26)

and

$$\left({\text{μ}}_{u},\log\;{\text{σ}}_{u}^{2})=h_{4}(c)=(M_{2}\cdot c,V_{2}\cdot c\right)$$ (27)

$$q(u_{\mathrm{ext}}|c)=N[u_{\mathrm{ext}}|{\text{μ}}_{u},\mathrm{diag}({\text{σ}}_{u}^{2})]$$ (28)

### Optimization

The optimization target is the negative dataset ELBO

$$L_{\mathrm{dataset}}=\sum_{i=1}^{n_{\mathrm{sub}}}\sum_{j=1}^{n}L_{ij}$$ (29)

where *L_ij_* is the ELBO associated with a subject *i* and region *j*, defined by Eqs. 10 to 12. We minimize the cost function over the weights of the LSTM networks *h*_1_, *h*_2_, weights of the neural network *f*, means and variances of the subject-specific parameters and of the external input time series **M**_1_, **M**_2_, **V**_1_, and **V**_2_; external input time scale τ, system; and observation noise variances ${\text{σ}}_{s}^{2}$ and $\sigma_{o}^{2}$ (in log scale), and forward projection parameters **A** and **b**.

The method is implemented in Keras 2.4 (*65*). The parameters of the method and of optimization procedure are given in table S1. For optimization, we use the Adam algorithm (*66*) while relying on the reparameterization trick (*67*) for backpropagation through the random node of the variational autoencoder. The expectations in Eqs. 10 to 12 are approximated using samples drawn from the approximate posterior distribution. For the validation on the synthetic data, the optimization is run for 2000 epochs with learning rate of 0.003 and then for additional 1000 epochs with learning rate of 0.001 with batch size of 16. For the application on resting-state fMRI data, the optimization is run for 1000 epochs with learning rate of 0.001 and batch size of 64. To make the optimization more stable, we use gradient clipping with limits (−1000 and 1000). To better guide the optimization procedure, we follow the previous works (*15*) with initial ELBO relaxation: The terms corresponding to the priors and approximate posteriors for states **x** and parameters **θ***^r^* and **θ***^s^* (Eqs. 11 and 12) are scaled by a coefficient β, which linearly increases from 0 to 1 between the 1st and 500th epoch.

Two regularization terms are added to the cost function. First is an L2 regularization on the kernel weight biases and of the neural network representing function *f*, $\alpha_{f}[\sum_{i=1}^{n_{w}}{\left(w_{i}^{f}\right)}^{2}+\sum_{i=1}^{n_{b}}{\left(b_{i}^{f}\right)}^{2}]$, where *n_w_* and *n_b_* is the number of kernel weights $w_{i}^{f}$ and bias coefficients $b_{i}^{f}$, respectively. Second is on the states **x**, $\alpha_{x}\sum_{i=1}^{n_{s}}\sum_{k=1}^{n_{s}}x_{k,i}^{2}$. We set α*_f_* = 0.01 and α*_x_* = 0.01.

The initial conditions for the optimization are set as follows. The log variances of the system noise are set to −2, and the log variances of the observation noise are set to 0. The projection vector **a** is initialized randomly drawing from normal distribution (mean, 0; SD, 0.3), and the projection bias *b* is set to zero. Matrices for subject-specific parameters and for external input **M**_1_, **M**_2_, **V**_1_, and **V**_2_ are initialized randomly drawing from a normal distribution (mean, 0; SD, 0.01). All layers of used neural networks use the default initialization provided by Keras.

To assess possible overfitting in the inference and generative networks, we adopt the following procedure when applying the method to resting-state fMRI data. We divide the time series from all regions and all subjects into the training set (80% of regions) and test set (20% of regions), and the optimization is then performed using the ELBO calculated from the training set only (fig. S11). We note that split is performed between the regional time series and not between subjects, that is, the regional time series in the test set come from the same subjects as the regional time series used for the training. This is a limitation of the present architecture, as it cannot be readily applied to new subjects because of the direct optimization of the external input **u**_ext_ and subject parameters **θ***^s^* and the encoding of the identity of the subject in one-hot vector **c**. Therefore, this procedure should not be understood as testing any predictive power for new dataset (we are making no claims in that regard), but rather evaluating if the generative model *f* is memorizing specific time series.

### Whole-brain network models for simulated datasets

#### Hopf bifurcation model

The Hopf model of large-scale brain dynamics (*8*) is built by placing a neural mass near supercritical Hopf bifurcation at each node of a brain network. Each neural mass *i* is described by two parameters: bifurcation parameter *a_i_* and intrinsic frequency *f_i_*. For *a_i_* < 0, the uncoupled neural mass has one stable fixed point, and for *a_i_* > 0, the neural mass has a stable limit cycle indicating sustained oscillations with frequency *f_i_*. The bifurcation exists at the critical value *a_i_* = 0. The dynamics of each node in the network are given by a set of two coupled nonlinear stochastic differential equations

$${\dot{x}}_{i}=(a_{i}-x_{i}^{2}-y_{i}^{2})x_{i}-\omega_{i}y_{i}+G\sum_{j=1}^{n}w_{ij}(x_{j}-x_{i})+{\beta \eta }_{i}^{x}(t)$$ (30)

$${\dot{y}}_{i}=(a_{i}-x_{i}^{2}-y_{i}^{2})y_{i}+\omega_{i}x_{i}+G\sum_{j=1}^{n}w_{ij}(y_{j}-y_{i})+{\beta \eta }_{i}^{y}(t)$$ (31)

where ω*_i_* = 2π*f_i_*, *G* > 0 is the scaling of the coupling, *w_ij_* is the weight of connection from node *j* to node *i*. Additive Gaussian noise η is included in the equations, with SD β.

To generate the synthetic dataset, we use the structural connectome matrices of the first eight subjects from the group described above. We simulate eight subjects, with increasing coupling coefficient *G* spaced linearly between 0 and 0.7. The intrinsic frequency *f_i_* of all nodes is sampled randomly from uniform distribution on [0.03, 0.07] Hz. The bifurcation parameter *a* is sampled randomly from uniform distribution [−1, 1]. The initial conditions of the system for all subjects and both variables are chosen randomly from normal distribution *N*(0, 0.3), and the system is then simulated for 205 s. The first 25 s are then discarded to avoid the influence of the initial conditions, leaving 180 s of data. The system is simulated with Euler method with time step Δ*_t_* = 0.02 s. As the observed variable, we take the first of the two variables in each node (i.e., *x_i_*), downsampled to 1 Hz; therefore, every time series contain 180 time points. The data are normalized to zero mean and variance equal to one (calculated across the whole dataset).

#### Parametric mean field model

The pMFM was derived as a reduction from a spiking neural model (*7*). The resulting model is described by one nonlinear stochastic differential equation in each node of the brain network

$${\dot{S}}_{i}=-\frac{S_{i}}{\tau_{s}}+(1-S_{i})\gamma H(x_{i})+\sigma \eta_{i}(t)$$ (32)

$$H(x_{i})=\frac{ax_{i}-b}{1-\exp\;[-d(ax_{i}-b)]}$$ (33)

$$x_{i}=r_{i}JS_{i}+I_{o}+I_{n}$$ (34)

where *x_i_* is the total input current, *H*(*x_i_*) is the population firing rate, and *S_i_* is the average synaptic gating variable. The total input current depends on the recurrent connection strength *r_i_*, synaptic coupling strength *J* = 0.2609 nA, excitatory subcortical input *I_o_* = 0.295 nA, and the regional coupling $I_{n}=G\sum_{j=1}^{n}w_{ij}S_{j}$, scaled by the global scaling coefficient *G*. The strength of the coupling between region *j* and *i* is proportional to the structural connection strength *w_ij_*. The kinetic parameters of the models are the decay time constant τ*_s_* = 100 ms and γ = 0.641/1000. Values for the input-output function *H*(*x_i_*) are *a* = 270nC^−1^, *b* = 108 Hz, and *d* = 0.154 s. Depending on the parameter values and the strength of the network coupling, the system can be either in monostable downstate regime at low firing rate values, bistable regime with two stable fixed points, or monostable upstate regime at high firing-rate values. The stochastic transitions between states are driven by the additive Gaussian noise η*_i_* with SD σ.

The initial conditions for *S_i_* were chosen randomly from uniform distribution on [0.2, 0.8]. The system was simulated for eight subjects with connectome matrices described above. For each subject, a specific value of coupling coefficient *G* producing the strongest FC was used. This was determined by performing 4-min-long simulations with subject-specific connectome and fixed regionally heterogeneous parameters, repeated for 31 values of *G* between 0.17 and 0.22 (where optimal value was expected to lie), and picking the value where the mean of FC from the last 2 min was the highest. With this value of *G*, the activity of each subject was simulated for 16.4 min, first two of which were discarded to avoid the influence of the initial conditions. The Euler method with time step Δ*_t_* = 10 ms was used for the simulation. The resulting time series of *S_i_* were temporally averaged over windows of size 0.72 s, leaving 1200 time points in every time series. The data are normalized to zero mean and variance equal to one (calculated across the whole dataset).

### Relation of regional parameters and regional features from individual and external data

To analyze the role of the regional parameters inferred from human resting-state fMRI data, we compare the inferred parameters to several regional features obtained on the individual level or on a population level from previous literature. All features are represented by a vector of 68 elements, corresponding to the cortical regions of Desikan-Killiany parcellation.

#### Features from individual data

The individual-level features are derived from the data used in the model fitting: structural connectivity and parcellated resting-state fMRI. For structural connectivity, it is node in-strength and eigenvector centrality. For fMRI data, it is the first and second spatial eigenvector obtained from PCA, vector of correlation coefficients of regional time series with the mean signal of resting-state fMRI, vector of correlation coefficients of regional time series with the network input time series (Eq. 7), number of zero-crossings of the regional signals, and the power below 0.1 Hz. We note that the signals are all normalized, so the total power is constant.

#### Features from external data

We further consider several regional features derived from other sources unrelated to the modeling data. First, it is the neuronal density and neuronal size derived from the pioneering work of Von Economo and Koskinas (*26*). These data were mapped to Desikan-Killiany atlas (*68*) and used previously in related large-scale brain modeling study (*6*). Our inspection of these remapped data however indicated possible error in the mapping, as it contained density values that would be obtained by summing across cortical sublayers instead of averaging. Therefore, we opted to redo the mapping using the tabular data given in the recent translation of the original work (*69*). We followed the procedure as described in (*68*), only taking an average of neuronal densities and sizes where multiple sublayers were given.

In addition, we have used neuronal density estimated from the BigBrain model (*27*). The BigBrain model is a 3D-reconstructed dataset with a spatial resolution of 20 μm isotropic, i.e., nearly cellular. The regional cell densities were estimated by randomly sampling at least 15 3D chunks per region from the cortex of the BigBrain model. For each region in the parcellation map, between 15 and 140 random coordinates were drawn in the ICBM 2009c nonlinear asymmetric space depending on the size of each region (*70*). Each coordinate was transformed to the BigBrain histological space using a nonlinear transformation guided by sulcal constraints (*71*) and then shifted to the closest position around the cortical mid-surface, as defined by the BigBrain layer segmentation (*72*). A square cube extending across the full cortical depth was sampled at each mid-surface location, and layer-specific histograms of BigBrain gray values were extracted from each cube. The gray values are 8-bit integers in the range of 0…255, where dark values indicate the presence of cell bodies due to the silver staining that was applied to the BigBrain tissue sections. To convert gray values into actual density estimates, a collection of 120 BigBrain cortical image patches scanned at 1-μm resolution [e.g., (*73*); full list of data references in data S1] with explicit layer-specific density estimates in numbers of segmented cell bodies (*74*) per 0.1 mm^3^ was used to compute an individual calibration function per layer. These calibration functions were applied to the mean gray value per layer in each sampled cube. From the resulting layerwise densities in each sampled cube, average cortical cell densities were determined by weighting each layer density with its relative volume. The final cell density estimate for each parcellation region was determined as the average cortical density across all cubes sampled from it.

Next, the RSFC principal gradient (*28*) was obtained from resting-state fMRI of large cohort of healthy adults by means of diffusion embedding, a nonlinear projection technique. It was proposed as a proxy measure for processing hierarchy. We use the numerical values provided by Kong *et al.* (*4*). The T1w/T2w ratio is used as a measure of myelin content. We use the map in Desikan-Killiany parcellation (*4*) obtained from the HCP dataset of 1200 healthy adults (*29*). Last, we consider two features derived from gene expression profiles (*5*) from the Allen Human Brain Atlas (*30*): First is the first principal component of 1926 brain-relevant genes. Second is the EI map, estimated from the expressions of genes coding for the excitatory AMPA and *N*-methyl-d-aspartate receptors and inhibitory GABA_A_ receptors.

#### Multivariate linear regression

All features were normalized before performing the linear regression. As the regional parameters **θ***^r^* were estimated probabilistically, that is, the mean and variance of a normal distribution were inferred, we performed the linear regression on 100 samples from these inferred distributions.

### Analysis of the inferred dynamical systems

To find the fixed points of the inferred dynamical systems we used the “hybr” root-finding method in SciPy package. We used multiple random initialization for each parameter configuration (60 for node-level analysis and 100 for network-level analysis), sampled uniformly from [−2, 2] in each dimension. The root-finding method used the system Jacobian calculated by TensorFlow’s automatic differentiation. Stopping tolerance was set to 1 × 10^−6^.

To assess whether the system supports unsteady dynamics (limit cycles or chaotic dynamics), we simulate the systems from the same random initializations for 288 s in the absence of system noise. After that, we evaluate whether the system has converged to a fixed point by criteria of the last 72 s being in the vicinity of the last final state (threshold 1 × 10^−3^ in L2 distance). If not, then we simulate the system further to 1200 s and evaluate again with the same criteria, and if the system has not converged, then it is marked as unsteady.

## Notes

View/request a protocol for this paper from *Bio-protocol*.
