# Supplementary material for: Characterization of regional differences in resting-state fMRI with a data-driven network model of brain dynamics
Source: Sci Adv. 2023 Mar 17;9(11):eabq7547. doi: 10.1126/sciadv.abq7547 (PMC10022900; doi:10.1126/sciadv.abq7547)
Supplement: Supplementary file 1 — Supplementary Text Table S1 and S2 Figs. S1 to S18 Legend for data S1 [file sciadv.abq7547_sm.pdf]

Science Advances
AAAS

Supplementary Materials for

Characterization of regional differences in resting-state fMRI with a data-driven network model of brain dynamics

Viktor Sip *et al.*

Corresponding author: Viktor Sip, vikt.sip@gmail.com; Viktor Jirsa, viktor.jirsa@univ-amu.fr

Sci. Adv. **9**, eabq7547 (2023)
DOI: 10.1126/sciadv.abq7547

The PDF file includes:

Supplementary Text
Tables S1 and S2
Figs. S1 to S18
Legend for data S1

Other Supplementary Material for this manuscript includes the following:

Data S1

Supplementary text

Evidence lower bound (ELBO)

For a single subject, the observations contain the time series from all n regions, $\boldsymbol{Y} = (\boldsymbol{y}_1, \ldots, \boldsymbol{y}_n) \in \mathbb{R}^{n\, n_t}$, where n_t is the number of time points. They are complemented by the region time series for the network input, $\boldsymbol{U} = (\boldsymbol{u}_1, \ldots, \boldsymbol{u}_n) \in \mathbb{R}^{n\, n_t}$, and the one-hot vector $\boldsymbol{c} \in \mathbb{R}^{n_{sub}}$ encoding the subject identity. The latent variables $\boldsymbol{Z}$ contain the state time series $\boldsymbol{x}_j \in \mathbb{R}^{n_s n_t}$ for all regions j (where n_s is the dimension of the state space), region-specific parameters $\boldsymbol{\theta}^r_j \in \mathbb{R}^{m_r}$, subject specific parameters $\boldsymbol{\theta}^s \in \mathbb{R}^{m_s}$, and time series of the external input $\boldsymbol{u}_{\text{ext}} \in \mathbb{R}^{n_t}$. The latent variables can be thus written as $\boldsymbol{Z} = (\boldsymbol{x}_1, \ldots, \boldsymbol{x}_n, \boldsymbol{\theta}^r_1, \ldots, \boldsymbol{\theta}^r_n, \boldsymbol{\theta}^s, \boldsymbol{u}_{\text{ext}})$. Our goal is to minimize the Kullback-Leibler divergence between the approximate and true posterior, which can be rewritten as a sum of subject ELBO and evidence itself,

$$\begin{aligned}
&\mathrm{KL}(q(\boldsymbol{Z}|\boldsymbol{Y}, \boldsymbol{U}, \boldsymbol{c}) \;||\; p(\boldsymbol{Z}|\boldsymbol{Y}, \boldsymbol{U}, \boldsymbol{c})) = \mathbb{E}_q[\log q(\boldsymbol{Z}|\boldsymbol{Y}, \boldsymbol{U}, \boldsymbol{c})] - \mathbb{E}_q[\log p(\boldsymbol{Z}|\boldsymbol{Y}, \boldsymbol{U}, \boldsymbol{c})] \\
&= \underbrace{\mathbb{E}_q[\log q(\boldsymbol{Z}|\boldsymbol{Y}, \boldsymbol{U}, \boldsymbol{c})] - \mathbb{E}_q[\log p(\boldsymbol{Y}|\boldsymbol{Z}, \boldsymbol{U}, \boldsymbol{c})] - \mathbb{E}_q[\log p(\boldsymbol{Z}|\boldsymbol{U}, \boldsymbol{c})]}_{-L_{\text{subject}}} + \mathbb{E}_q[\log p(\boldsymbol{Y}|\boldsymbol{U}, \boldsymbol{c})].
\end{aligned}$$

Maximizing the ELBO then minimizes the KL divergence. We can factorize all terms of the ELBO across n brain regions: the approximate posterior,

$$q(\boldsymbol{Z}|\boldsymbol{Y}, \boldsymbol{U}, \boldsymbol{c}) = \prod_{j=1}^{n} q(\boldsymbol{x}_j|\boldsymbol{y}_j, \boldsymbol{u}_j, \boldsymbol{c}) \prod_{j=1}^{n} q(\boldsymbol{\theta}^r_j|\boldsymbol{y}_j, \boldsymbol{u}_j, \boldsymbol{c})\, q(\boldsymbol{\theta}^s|\boldsymbol{c})\, q(\boldsymbol{u}_{\text{ext}}|\boldsymbol{c}),$$

the data likelihood,

$$p(\boldsymbol{Y}|\boldsymbol{Z}, \boldsymbol{U}, \boldsymbol{c}) = \prod_{j=1}^{n} p(\boldsymbol{y}_j|\boldsymbol{x}_j, \boldsymbol{\theta}^r_j, \boldsymbol{\theta}^s, \boldsymbol{u}_{\text{ext}}, \boldsymbol{u}_j, \boldsymbol{c}),$$

and the prior,

$$\begin{aligned}
p(\boldsymbol{Z}|\boldsymbol{U}, \boldsymbol{c}) &= p(\boldsymbol{x}_1, \ldots, \boldsymbol{x}_n|\boldsymbol{\theta}^r_1, \ldots, \boldsymbol{\theta}^r_n, \boldsymbol{\theta}^s, \boldsymbol{u}_{\text{ext}}, \boldsymbol{U}, \boldsymbol{c})\; p(\boldsymbol{\theta}^r_1, \ldots, \boldsymbol{\theta}^r_n, \boldsymbol{\theta}^s, \boldsymbol{u}_{\text{ext}}|\boldsymbol{U}, \boldsymbol{c}) \\
&= \prod_{j=1}^{n} p(\boldsymbol{x}_j|\boldsymbol{\theta}^r_j, \boldsymbol{\theta}^s, \boldsymbol{u}_{\text{ext}}, \boldsymbol{u}_j, \boldsymbol{c}) \prod_{j=1}^{n} p(\boldsymbol{\theta}^r_j|\boldsymbol{u}_j, \boldsymbol{c})\, p(\boldsymbol{\theta}^s|\boldsymbol{c})\, p(\boldsymbol{u}_{\text{ext}}|\boldsymbol{c}).
\end{aligned}$$

We require that the data likelihood and priors depend on the subject identity only through the latent variables, so we remove the dependence on $\boldsymbol{c}$. We also require that the priors of $\boldsymbol{\theta}^r_j$ do not depend on the external input $\boldsymbol{u}_j$. Then we define the region ELBO as

$$\begin{aligned}
L_j = &\; \mathbb{E}_q[\log p(\boldsymbol{y}_j|\boldsymbol{x}_j, \boldsymbol{\theta}^r_j, \boldsymbol{\theta}^s, \boldsymbol{u}_{\text{ext}}, \boldsymbol{u}_j)] \\
&+ \mathbb{E}_q[\log p(\boldsymbol{x}_j|\boldsymbol{\theta}^r_j, \boldsymbol{\theta}^s, \boldsymbol{u}_{\text{ext}}, \boldsymbol{u}_j)] + \mathbb{E}_q[\log p(\boldsymbol{\theta}^r_j)] + \frac{1}{n}\mathbb{E}_q[\log p(\boldsymbol{\theta}^s)] + \frac{1}{n}\mathbb{E}_q[\log p(\boldsymbol{u}_{\text{ext}})] \\
&- \mathbb{E}_q[\log q(\boldsymbol{x}_j|\boldsymbol{y}_j, \boldsymbol{u}_j, \boldsymbol{c})] - \mathbb{E}_q[\log q(\boldsymbol{\theta}^r_j|\boldsymbol{y}_j, \boldsymbol{u}_j, \boldsymbol{c})] - \frac{1}{n}\mathbb{E}_q[\log q(\boldsymbol{\theta}^s|\boldsymbol{c})] - \frac{1}{n}\mathbb{E}_q[\log q(\boldsymbol{u}_{\text{ext}}|\boldsymbol{c})] \qquad (1)
\end{aligned}$$

so that the subject ELBO is the sum of region ELBOs, $L_{\text{subject}} = \sum_{j=1}^{n} L_j$.

	Hopf model validation	pMFM validation	Application on HCP data
State space dimension n_s	2	2	3
Number of region-specific parameters m_r	2	2	$3^{\dagger}$
Number of subject-specific parameters m_s	1	1	0
Number of hidden units in f	32	32	32
Number of LSTM units in h_1 and h_2	32	32	32
Batch size	16	16	64
Samples over the approximate posterior	8	8	8
External input	No	No	Yes
Number of subjects	8	8	100
Number of regions	68	68	68
Time series length n_t	180	1200	1200
Number of model parameters	18737	18737	19128
Number of additional parameters per subject	514	514	2914

Table S1: Method parameters used in the test cases on synthetic data and for the application on HCP resting-state fMRI data. †Or varying.

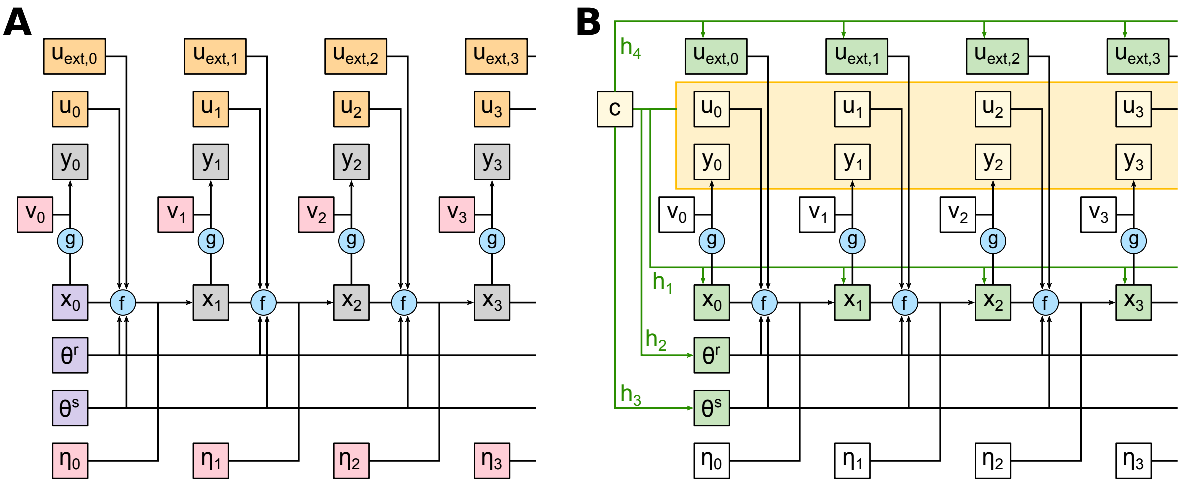

Figure S1: Overview of the method architecture visualized for one brain region. In the sketches we drop the region indices for simplicity, and keep only the time indices. (A) Generative model. With known functions f and g, and given initial conditions $\boldsymbol{x}_0$, parameters $\boldsymbol{\theta}^r$ and $\boldsymbol{\theta}^s$ and time-varying external input u_{ext}, the model can be simulated in forward fashion, influenced by the system noise $\boldsymbol{\eta}$ and observation noise ν. The network input for region j at time k is calculated on the fly from the current states of other regions, $u_{j,k} = \sum_{i=1}^{n} w_{ji} y_{i,k}$. (B) Inference model. The data (observation time series $\boldsymbol{y}$, precomputed network input time-series $\boldsymbol{u}$ and one-hot vector $\boldsymbol{c}$ identifying the subject) are mapped through the encoder functions h_1, h_2, h_3, and h_4 onto the system states $\boldsymbol{x}$, region-specific parameters $\boldsymbol{\theta}^r$, subject-specific parameters $\boldsymbol{\theta}^s$, and external input u_{ext} respectively. The observation function g appears in the likelihood function, while the system evolution function f enters the prior on the states. The noise $\boldsymbol{\eta}$ and ν is present only implicitly via the likelihood and the prior functions. The inference problem amounts to the maximization of the resulting ELBO over the parameters of the generative model f, g, encoder functions h_1, h_2, h_3, h_4, and variance of the system and observation noise.

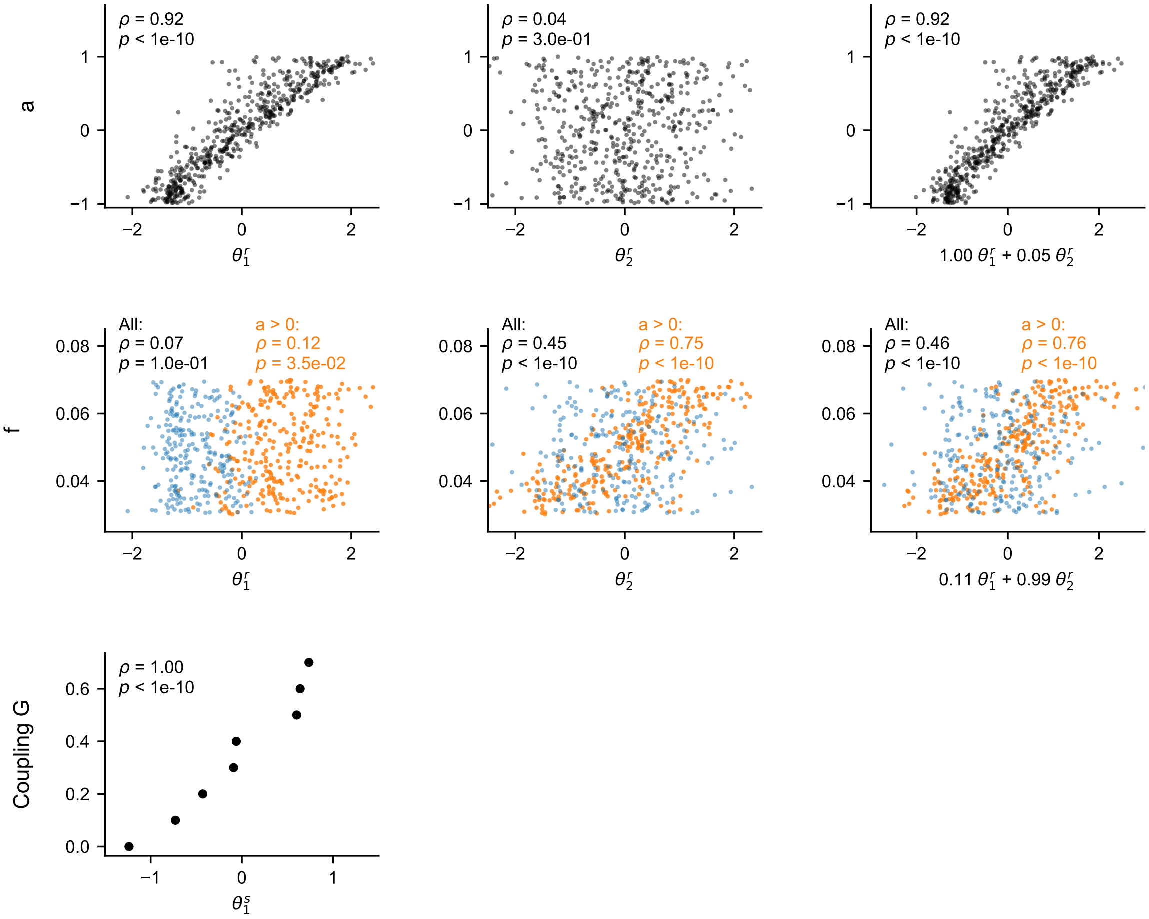

Figure S2: Relation of the original model parameters with inferred parameters for the Hopf model testcase, providing illustration for the statistics in Fig. 4A in the main text. The three rows shows the bifurcation parameter a, frequency f, and coupling coefficient G. In the upper two rows, each point represents a parameter of one region ($n = 544$, 8 subjects each with 68 regions), in the bottom row each point represents one subject ($n = 8$). The visualised data points shows a single sample from a posterior distribution. The text indicates Spearman's nonlinear correlation coefficient ρ. In the upper two rows, the rightmost panel shows the optimal projection of the parameters identified by multivariate linear regression of the posterior means. In the center row, data points are colored based on the original value of the bifurcation parameter a and Spearman's ρ is reported for both the full data set and the subset for $a > 0$.

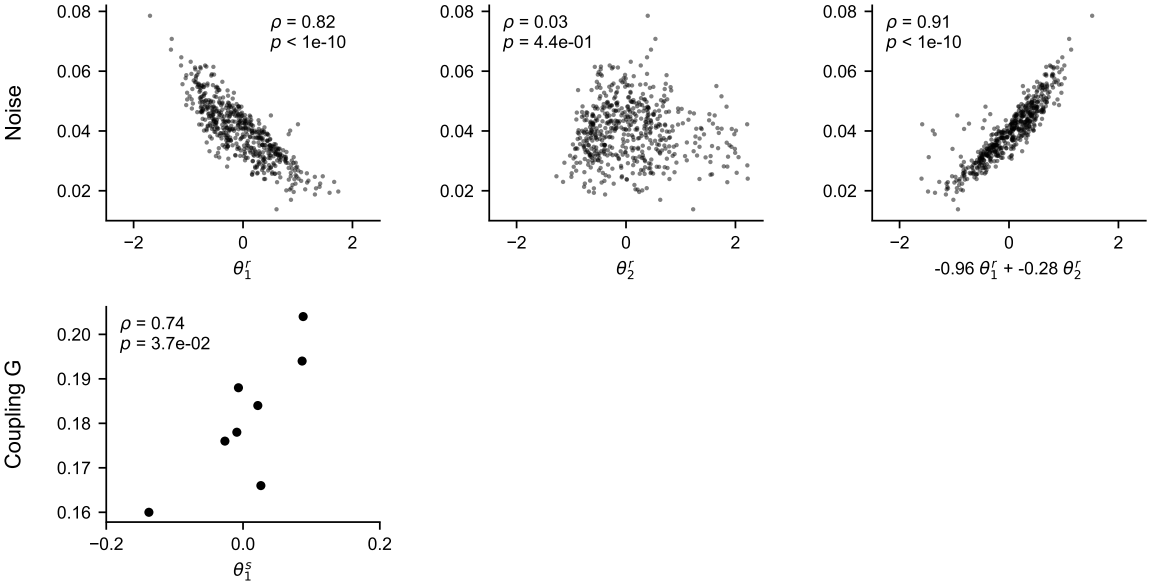

Figure S3: Relation of the original model parameters with inferred parameters for the pMFM testcase, providing illustration for the statistics in Fig. reffig:evalE in the main text. The two rows shows the noise strength and coupling coefficient G. In the upper row, each point represents a parameter of one region ($n = 544$, 68 regions and 8 subjects), in the bottom row each point represents one subject ($n = 8$). The visualised data points shows a single sample from a posterior distribution. The text indicates Spearman's nonlinear correlation coefficient ρ. In the upper row, the rightmost panel shows the optimal projection of the parameters identified by multivariate linear regression of the posterior means.

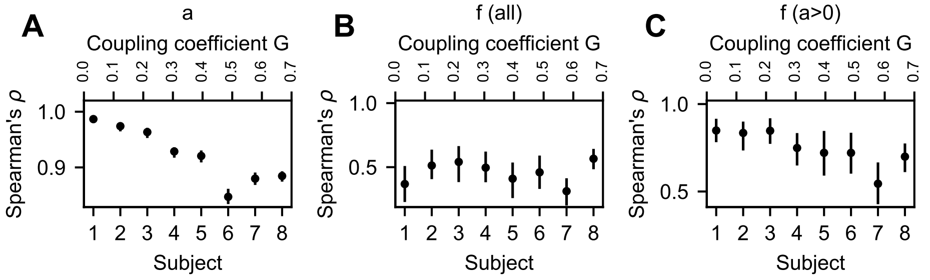

Figure S4: Recovery of the region-specific parameters in the Hopf model for different subjects with different coupling coefficient G. The figure contains the data from Fig. 2A in the main text, separated for the individual subjects.

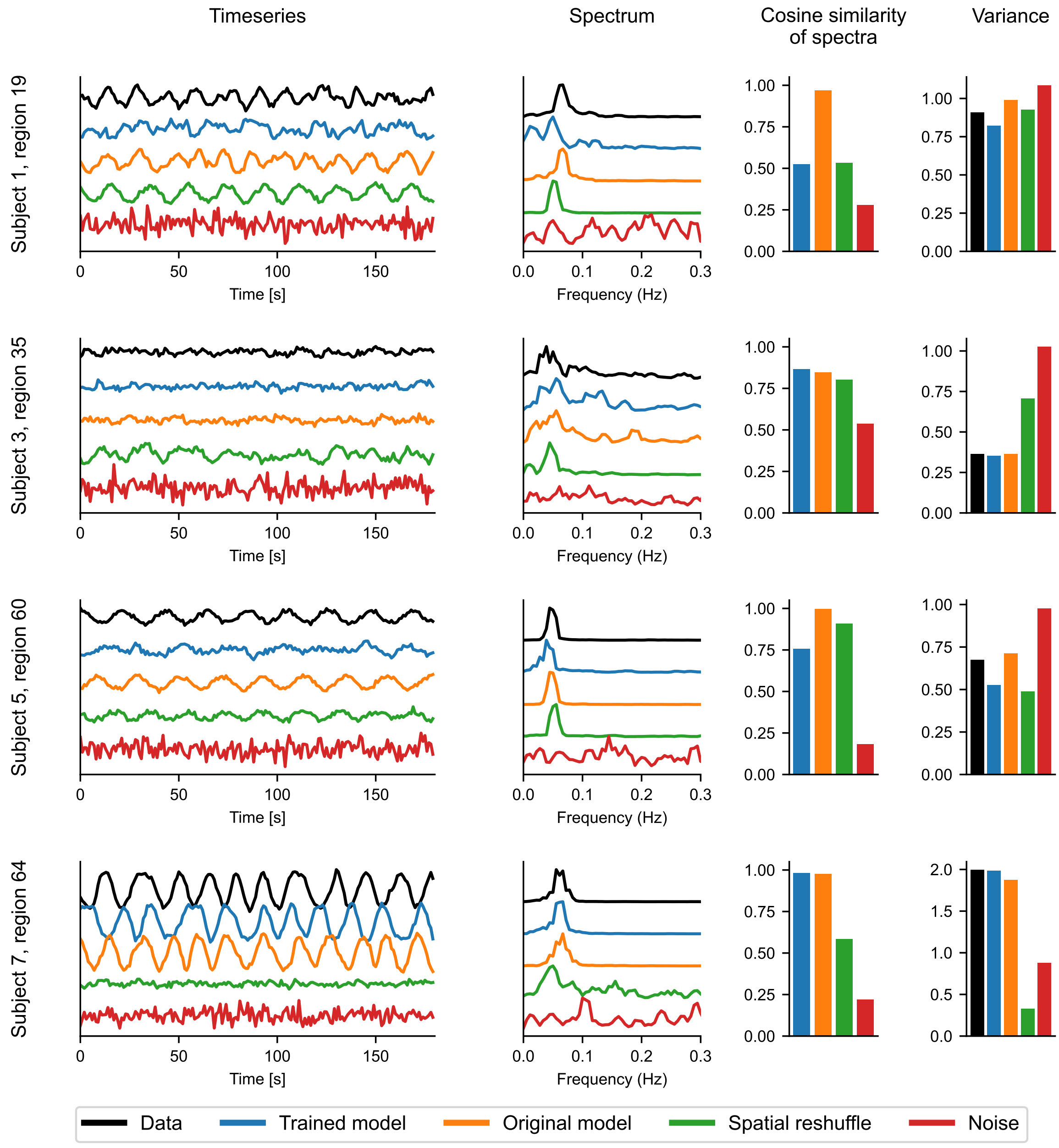

Figure S5: Examples of several reconstructed time series from the Hopf model testcase, and of the quantities used for the evaluation of the reconstruction quality on Fig. 4 in the main text: cosine similarity of the spectra and signal variance. Apart from the original data and an example reconstruction from the trained model, three surrogates are shown: new data generated with the original model, spatial reshuffle, and random noise. The spectra are normalized by its maximum for visualization purposes.

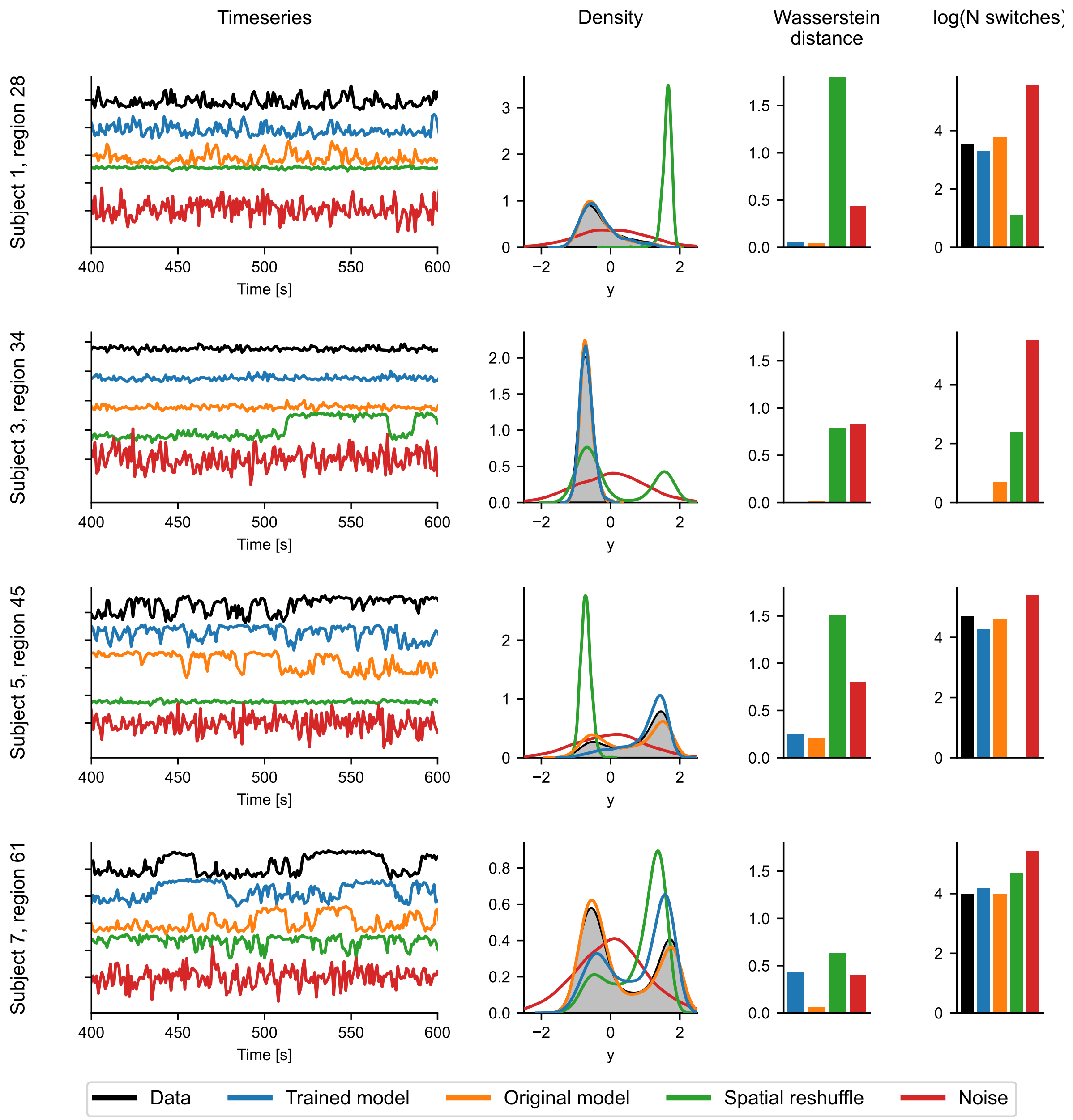

Figure S6: Examples of several reconstructed time series from the pMFM testcase, and of the quantities used for the evaluation of the reconstruction quality on Fig. 4 in the main text: Wasserstein distance of the probability densities, and number of switches on log-scale (bounded by 0). Apart from the original data and an example reconstruction from the trained model, three surrogates are shown: new data generated with the original model, spatial reshuffle, and random noise. In the leftmost panels, only 200 out of 1200 seconds of the timeseries is shown for visual clarity. In the center panel, the probability density of the original data is shaded.

**Hopf model, cosine similarity of spectra**					
	P_5	Median	P_{95}	Mean	p-value
Trained model	0.578	0.750	0.908	0.739	-
Original model	0.749	0.904	0.998	0.892	2.4e-103
Spatial reshuffle	0.282	0.708	0.977	0.672	1.2e-09
Noise	0.118	0.319	0.701	0.362	2.5e-184
**Hopf model, difference in variance**					
	P_5	Median	P_{95}	Mean	p-value
Trained model	0.011	0.130	0.680	0.211	-
Original model	0.010	0.059	0.172	0.070	6.2e-49
Spatial reshuffle	0.019	0.761	3.196	1.106	4.5e-71
Noise	0.112	0.784	2.329	0.885	4.2e-94
**pMFM, Wasserstein distance**					
	P_5	Median	P_{95}	Mean	p-value
Trained model	0.009	0.029	0.307	0.077	-
Original model	0.034	0.064	0.345	0.110	4.1e-08
Spatial reshuffle	0.041	0.597	2.371	0.919	5.7e-85
Noise	0.286	0.752	1.789	0.824	1.2e-155
**pMFM, difference in number of switches**					
	P_5	Median	P_{95}	Mean	p-value
Trained model	0.000	0.272	1.587	0.480	-
Original model	0.000	0.243	1.290	0.393	1.8e-03
Spatial reshuffle	0.000	1.946	4.651	2.047	7.7e-72
Noise	0.736	4.348	5.529	3.754	3.0e-153

Table S2: Numerical values and results of statistical testing for data presented in Fig. 4 in the main text. The columns show the 5-percentile, median, 95-percentile, mean of the distribution, and the p-value from comparison with the data from the trained model (two-sided t-test for related samples).

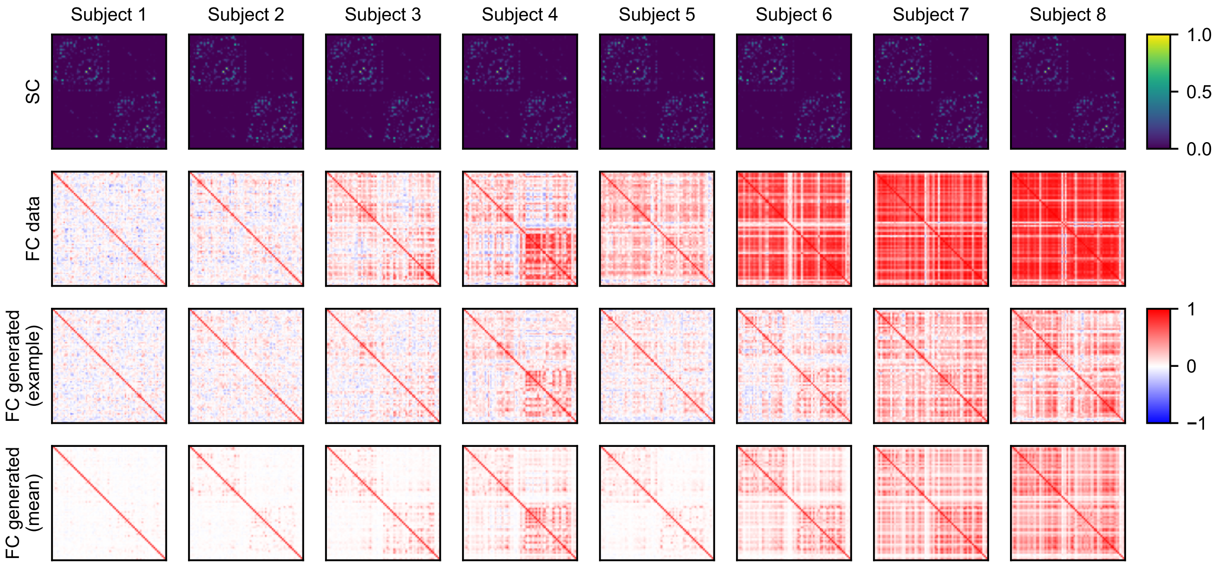

Figure S7: Structural and functional connectivity matrices for all subjects in the Hopf model test case. First row: structural connectivity. Second row: Functional connectivity of the original data used for the training. Third row: Functional connectivity of the example data generated with the trained model. Fourth row: Functional connectivity of the data generated with the trained model, averaged over 50 samples.

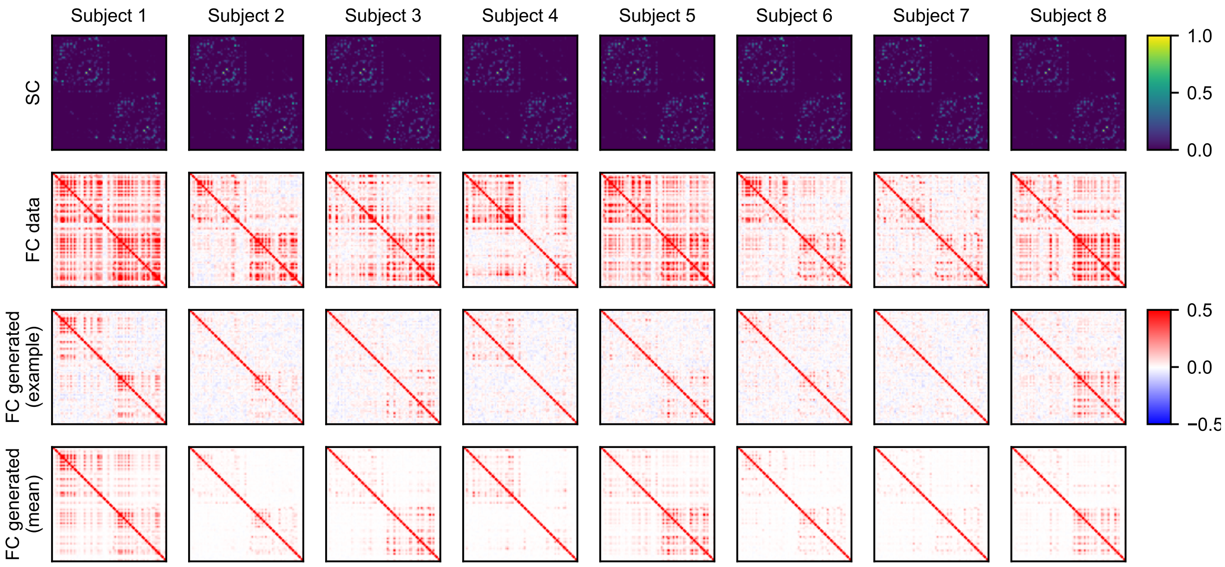

Figure S8: Structural and functional connectivity matrices for all subjects in the pMFM test case. Layout the same as in Fig. S7

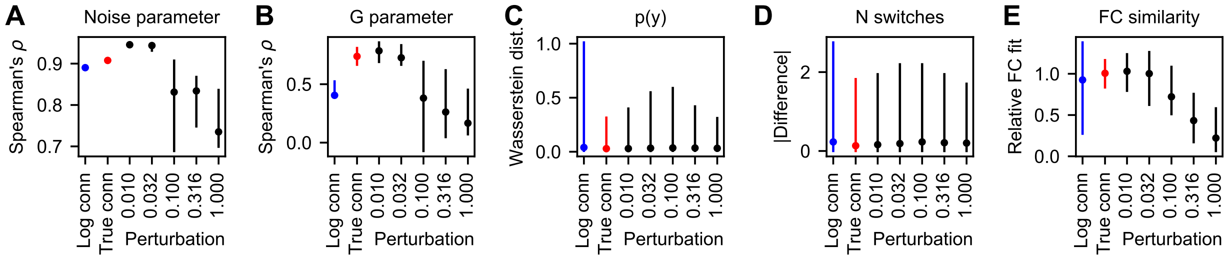

Figure S9: Effect of the connectome perturbation. (A) Spearman's correlation coefficient for the recovery of the noise parameter for the log-scaled connectome (blue), original connectome (red), and five perturbed connectomes (black). (B) Spearman's correlation coefficient for the recovery of the coupling parameter G. (C) Wasserstein distance of the distributions in the observation space of the original data and the data generated by the trained model. (D) Difference of the logarithm of number of switches between down- and up-state of the regional timeseries. (E) Relative FC fit, that is, normalized Pearson's correlation coefficient between the non-diagonal elements of the original FC and the FC generated by the trained model. The normalization is performed by dividing the coefficients by the mean of values obtained for the true connectome for every subject separately. The normalization is done in order to make the values comparable across subjects. For all panels, data were generated using four different connectome perturbations for each magnitude value, and one connectome for the original and log-scaled connectome. In panels A and B, 100 samples were drawn from the parameter distributions for each trained model. In panels C-E 50 simulations were performed to calculate the measures of goodness-of-fit for each model. These were then aggregated across all subjects (and across regions apart from panels B and E). Each line represents the 5 to 95 percentile range, with the dot representing the median.

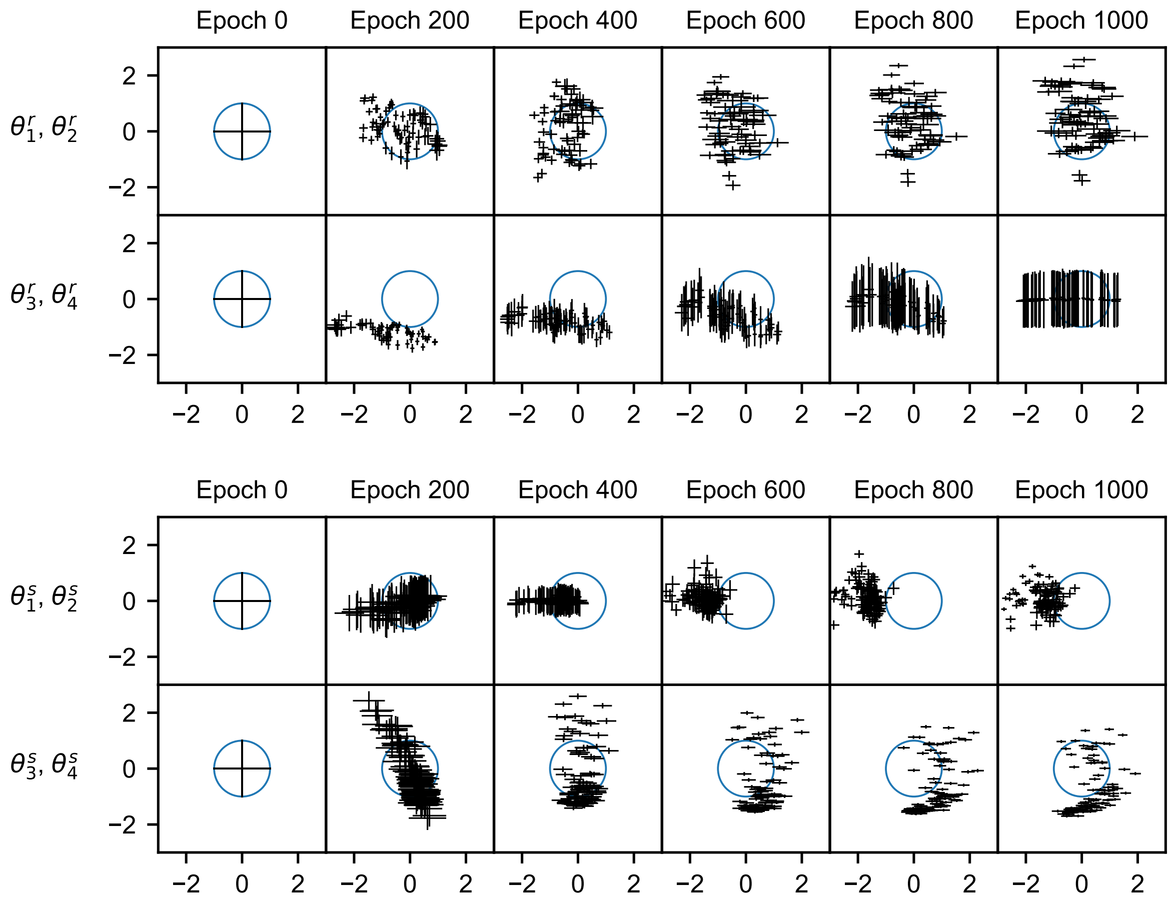

Figure S10: Illustration of the convergence of the regional and subject parameters during training on 100 subject data set from Human Connectome Project. The upper two rows show four out of five regional parameters during 1 000 epochs for a model with $m_r = 5$ and $m_s = 0$. Each cross represents the means and standard deviations of the inferred parameters of one region; for clarity, parameters of only one subject out of hundred are shown. The lower two rows show four out of five subject parameters for a model with $m_r = 3$ and $m_s = 5$. Each cross correspond to parameters of one subject. Blue circle represents the prior standard normal distribution. As expected and desired with variational autoencoders, the regional parameters after the training are roughly covering the support of the prior distribution, and in some unused dimensions (here θ_4^r) are closely matching the prior distribution $N(0, 1)$, exhibiting the posterior collapse phenomenon. Such behavior is however not observed for the subject parameters, which neither fully cover the prior distribution, nor do they exhibit posterior collapse in any dimension. While these inferred subject parameters might contain some information about the intersubject differences (as in the synthetic test cases on Fig. 2 and Fig. 3), this convergence failure limits their interpretability, as it is not guaranteed that non-collapsed parameters have any effect on the dynamics.

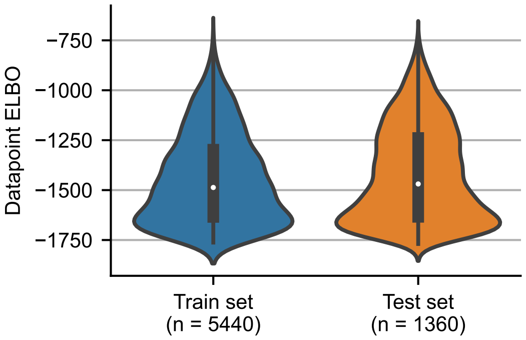

Figure S11: Assessment of the overfitting of the model trained on the Human Connectome Project data set. When training the model, all 100 subjects are used, but randomly selected 20% of regional time series are left out to assess the possible overfitting. To do so, we evaluate the datapoint ELBO (Eg. 1 in this SI) for the regions in the train and test sets. The overlapping distributions indicate that overfitting is not a major issue.

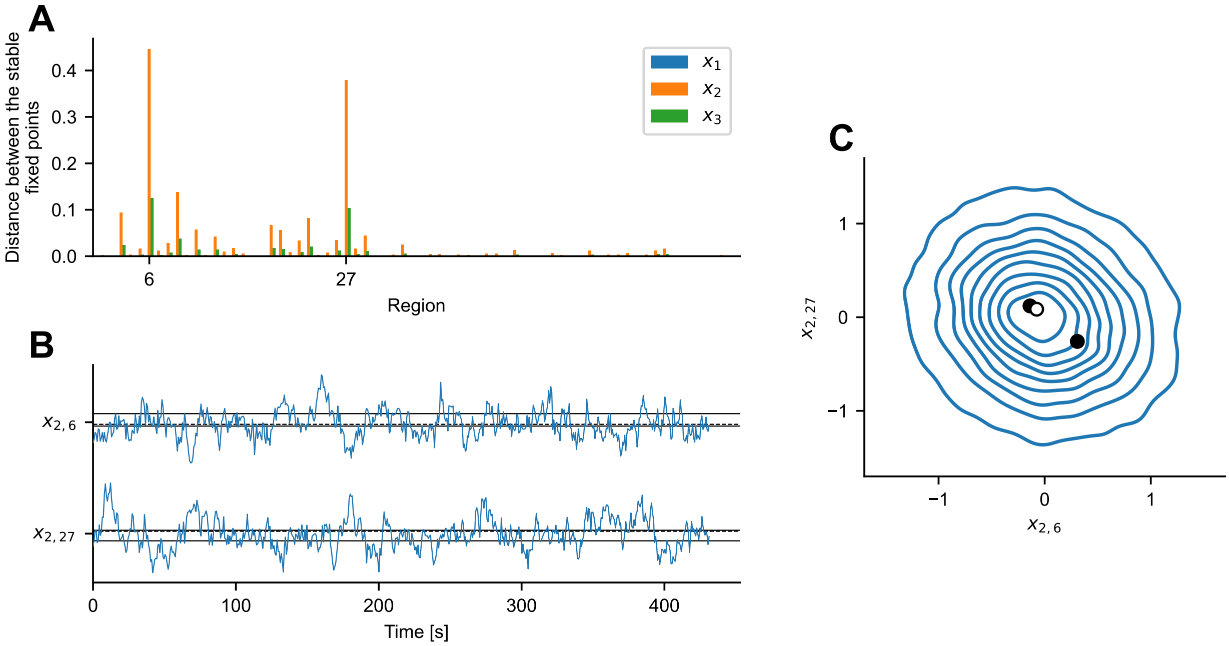

Figure S12: Example of a subject with discovered multistability. Subject 45 was among the six subjects having multiple stable fixed points (Fig.6). The search for the fixed points discovered two stable and one unstable fixed points. (A) Distance between the stable fixed points in the state space. The fixed points were separated mainly in the state space of region 6 and 27, on which we focus in the following panels. (B) Simulated activity in the two selected nodes. The full lines indicate the position of the stable fixed points, the dashed line the position of the unstable fixed point. (C) Probability density of the states of the two example regions across time. The stable fixed points are represented by the black-faced circles, the unstable with the white-faced circle. Neither from the panel B or C does the existence of the multiple stable fixed point seem to affect the generated activity, as no multimodality is immediately visible; the presence of the noise play the dominant role.

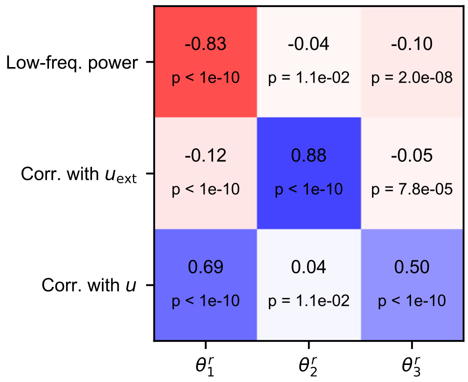

Figure S13: Results of the multivariate regression between the regional parameters and three timeseries features from Fig. 7 in the main text. In each cell, upper row gives the weight of the parameter, the bottom row the p-value.

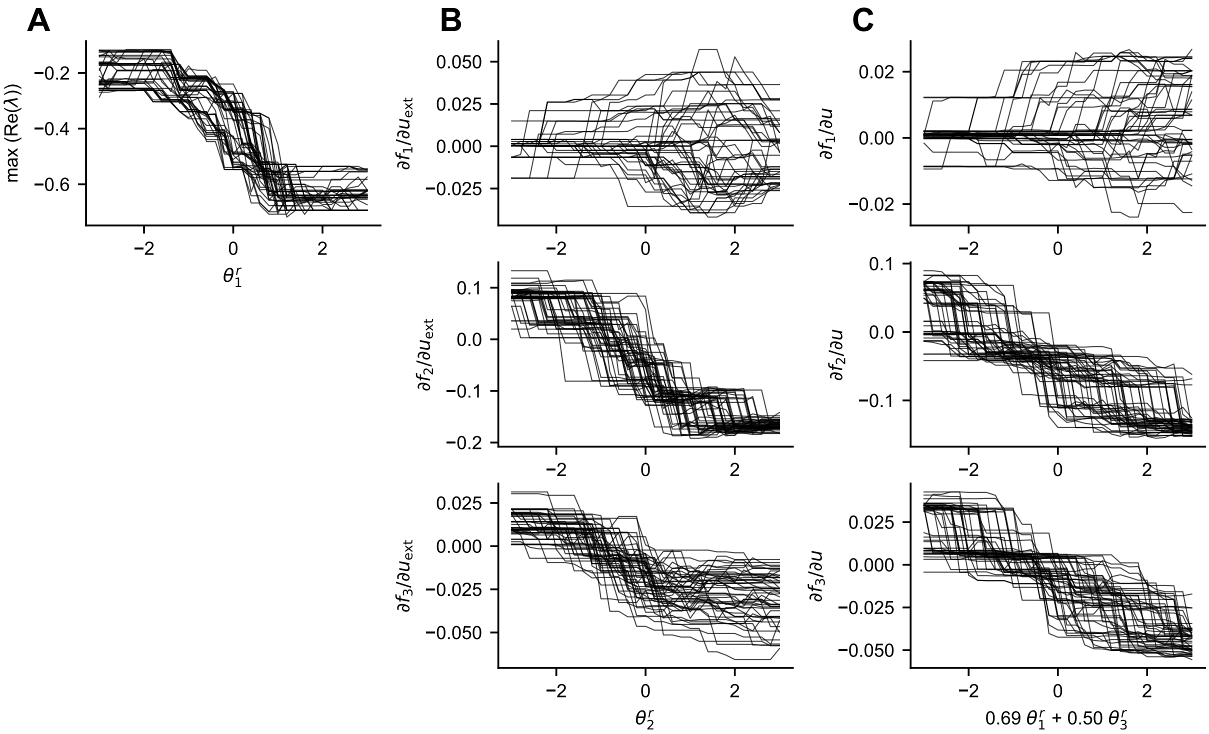

Figure S14: Analysis the role of the regional parameters $\boldsymbol{\theta}^r$ in the dynamical system $\dot{\boldsymbol{x}}(t) = f\left(\boldsymbol{x}(t), \boldsymbol{\theta}^r, \boldsymbol{\theta}^s, u_{\text{ext}}(t), u(t)\right) + \boldsymbol{\eta}(t)$, supporting Fig. 7 in the main text. In each panel, we show how a quantity of interest changes while traversing the parameter space. (A) Largest eigenvalue of the system at the fixed point. (B) Partial derivative of the three components of function f w.r.t. the external input u_{ext}. (C) Partial derivative of the three components of function f w.r.t. the network input u. In order to demonstrate that the hypothesised effects of the parameters hold across the whole parameter and state space, each line correspond to one of 50 random draws of the state $\boldsymbol{x} \sim U(-2, 2)$, regional parameters $\boldsymbol{\theta}^r \sim U(-2, 2)$, external input $u_{\text{ext}} \sim U(-2, 2)$, and network input $u \sim U(-2, 2)$. Only the parameter indicated on the horizontal axis is varied. Panel (A) demonstrates that increasing θ_1^r reduces the largest eigenvalue, thus accelerating the decay towards the fixed point, leading to the reduction of noise-induced low-frequency oscillations. Panel (B) shows that θ_2^r modulates the response of the dynamical system to the external input. Panel (C) similarly shows that how the linear combination of θ_1^r and θ_3^r (Fig. S13) modulates the response to the network input.

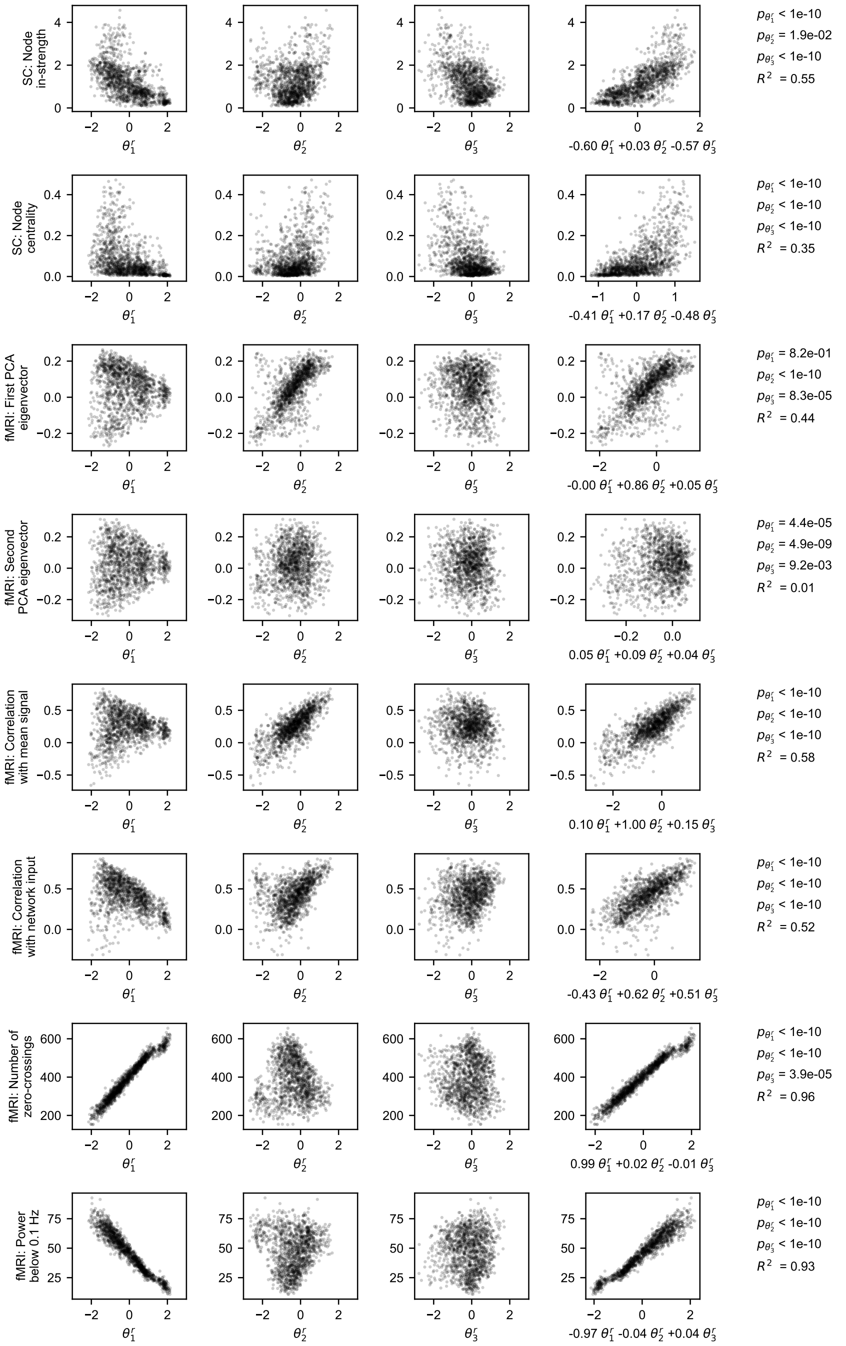

Figure S15: Human resting-state fMRI: Relation between the inferred parameters and features from individual data. Each dot corresponds to one region of one subject. The position corresponds to the inferred mean of regional parameters, the variances are not visualized. The fourth column shows the optimal direction identified by the multivariate linear regression (as in Tab. 2). In the rightmost column are the p-values from the multivariate linear regression (two-sided t-test, uncorrected values) and R^2 coefficient of determination. The analysis is performed on $n = 6800$ data points (100 subject, 68 regions each). For the purpose of visualization only, the number of data point is randomly downsampled by a factor of 5 (6800 to 1360).

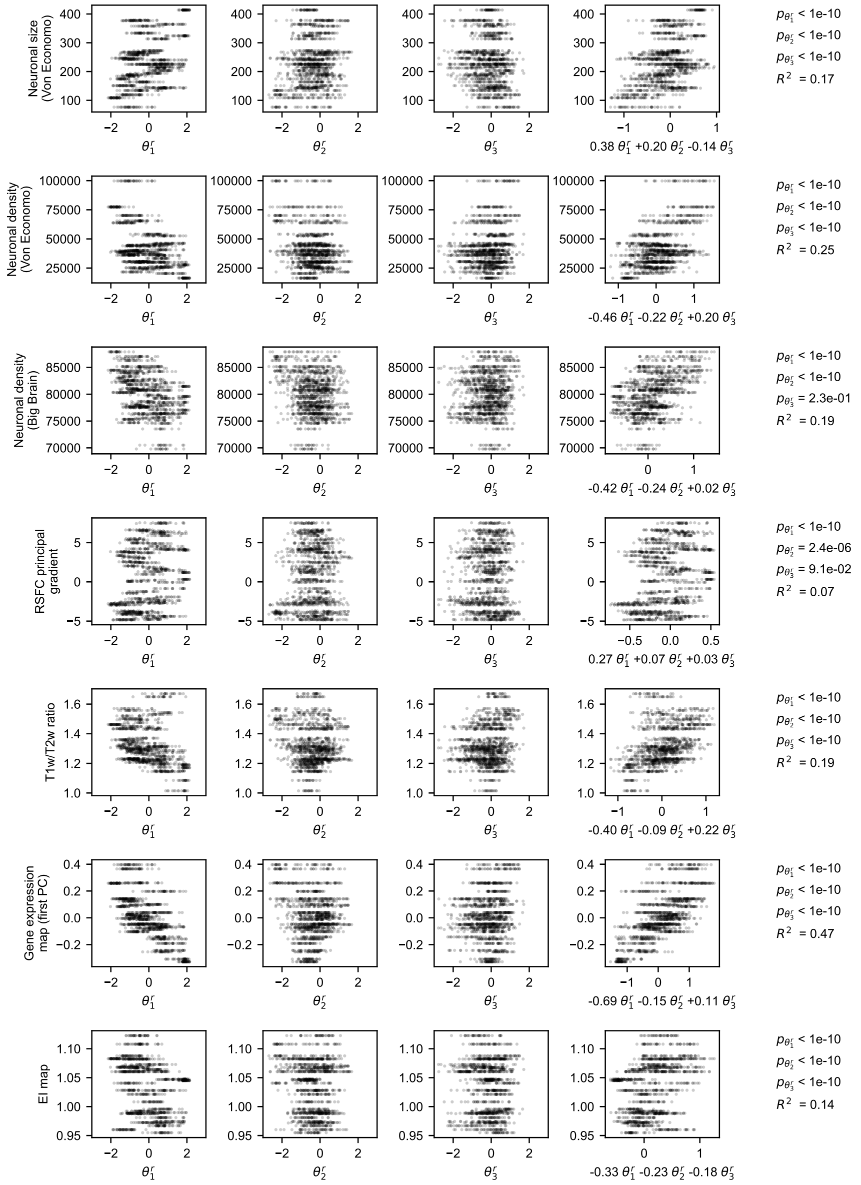

Figure S16: Human resting-state fMRI: Relation between the inferred regional parameters and features from external data. Each dot corresponds to one region of one subject. The position corresponds to the inferred mean of regional parameters, the variances are not visualized. The fourth column shows the optimal direction identified by the multivariate linear regression (as in Tab. 2). In the rightmost column are the p-values from the multivariate linear regression (two-sided t-test, uncorrected values) and R^2 coefficient of determination. The analysis is performed on $n = 6800$ data points (100 subject, 68 regions each). For the purpose of visualization only, the number of data point is randomly downsampled by a factor of 5 (6800 to 1360). The horizontal bands are caused partly by the limited precision of the data obtained from other studies, and partly by the fact that the feature maps of 68 regions are not individual, therefore there are at most 68 different values on the vertical axis.

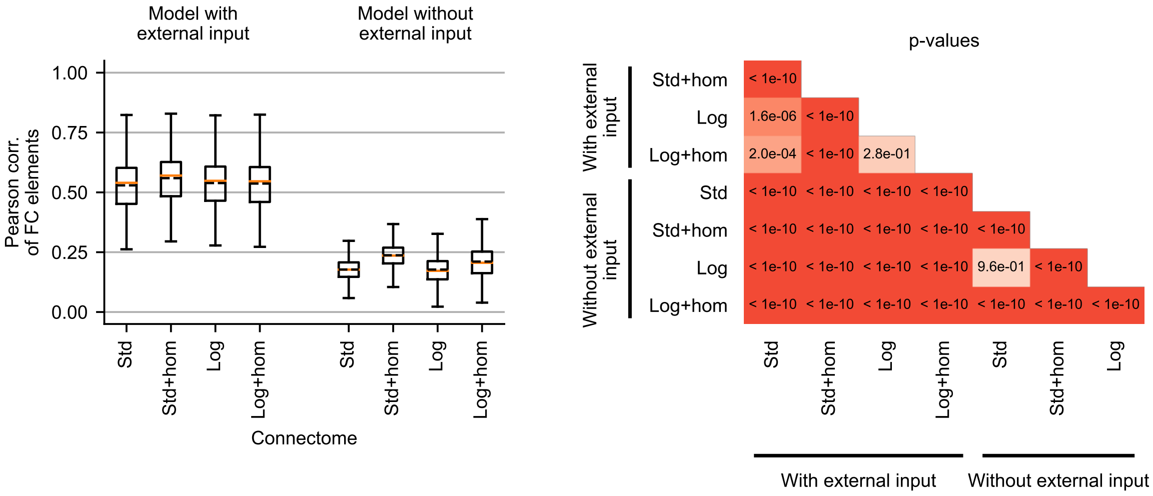

Figure S17: Effect of the modified connectome on the generated functional connectivity. The fit is evaluated in terms of similarity of functional connectivity, measured by the Pearson correlation of the FC elements. Results are shown for the variants without and with external input, and for four variants of structural connectomes: standard (Std), standard with homotopic connections added (Std+hom), log-scaled (Log), and log-scaled with homotopic connections added (Log+hom). Each boxplot is constructed from $n = 2000$ data points (100 subjects, 20 simulations each). The box extends from the first quartile to the third quartile of the data, with a solid line at the median and dashed line at mean. The whiskers extend from the box by 1.5x the inter-quartile range. Fliers are not shown. Right panel shows the p-values (two-sided t-test, not corrected for multiple comparison) between all variants.

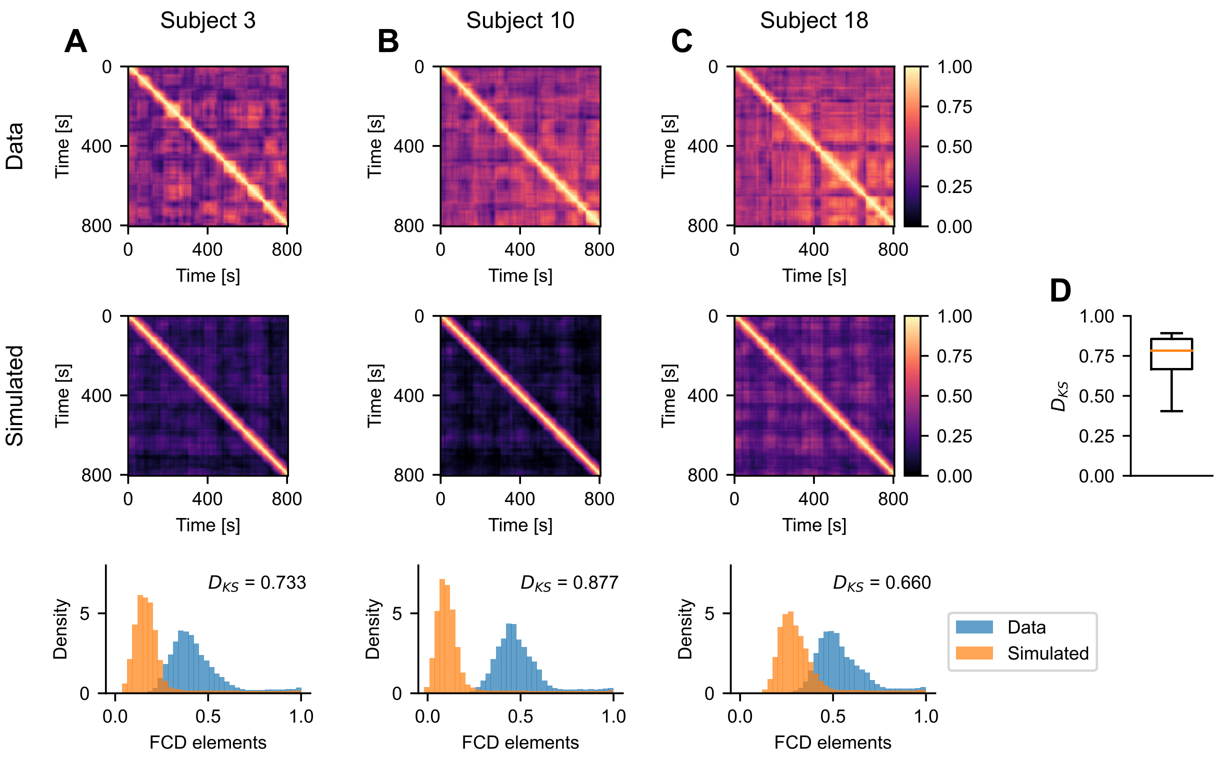

Figure S18: Functional connectivity dynamics (FCD) in the original and simulated data. FCD refers to the time-varying changes in functional connectivity; we quantify and visualize these changes with the FCD matrices, in which the (i,j) element is the Pearson correlation between the functional connectivity matrices at times i and j. These FC matrices are calculated with a sliding window of 60 seconds. (A-C) Three examples, showing the original FCD (upper panel), FCD in the simulated data (middle panel), and the distributions of FCD elements in the original and simulated data, and the Kolgomorov-Smirnov distance D_{KS} between them. Perfect overlap of the two distributions, and D_{KS} close to zero would indicate that the FCD structure is well reproduced; this is however not the case in the examples. (D) Evaluation of FCD fit for all subjects, quantified with the Kolgomorov-Smirnov distance. The box extends from the first quartile to the third quartile of the data, with a solid line at the median. The values far from zero indicate that the model is not capable of reproducing the FCD structure well.

Data S1. (separate file)
Data references on EBRAINS portal for layer-specific distributions of segmented cells in the BigBrain model.
